# KIBRA repairs synaptic plasticity and promotes resilience to tauopathy-related memory loss

**DOI:** 10.1172/JCI169064

**Published:** 2024-02-01

**Authors:** Grant Kauwe, Kristeen A. Pareja-Navarro, Lei Yao, Jackson H. Chen, Ivy Wong, Rowan Saloner, Helen Cifuentes, Alissa L. Nana, Samah Shah, Yaqiao Li, David Le, Salvatore Spina, Lea T. Grinberg, William W. Seeley, Joel H. Kramer, Todd C. Sacktor, Birgit Schilling, Li Gan, Kaitlin B. Casaletto, Tara E. Tracy

**Affiliations:** 1 Buck Institute for Research on Aging, Novato, California, USA.; 2 Memory and Aging Center, Department of Neurology, University of California San Francisco, San Francisco, California, USA.; 3 Gladstone Institutes, San Francisco, Califoria, USA.; 4 Weill Institute for Neurosciences, Department of Pathology, University of California San Francisco, San Francisco, California, USA.; 5 The Robert F. Furchgott Center of Neural and Behavioral Science, Departments of Physiology and Pharmacology, Anesthesiology, and Neurology, State University of New York Health Sciences University, Brooklyn, New York, USA.; 6 Helen and Robert Appel Alzheimer Disease Research Institute, Brain and Mind Research Institute, Weill Cornell Medicine, New York, New York, USA.

**Keywords:** Neuroscience, Alzheimer disease, Memory, Synapses

## Abstract

Synaptic plasticity is obstructed by pathogenic tau in the brain, representing a key mechanism that underlies memory loss in Alzheimer’s disease (AD) and related tauopathies. Here, we found that reduced levels of the memory-associated protein KIdney/BRAin (KIBRA) in the brain and increased KIBRA protein levels in cerebrospinal fluid are associated with cognitive impairment and pathological tau levels in disease. We next defined a mechanism for plasticity repair in vulnerable neurons using the C-terminus of the KIBRA protein (CT-KIBRA). We showed that CT-KIBRA restored plasticity and memory in transgenic mice expressing pathogenic human tau; however, CT-KIBRA did not alter tau levels or prevent tau-induced synapse loss. Instead, we found that CT-KIBRA stabilized the protein kinase Mζ (PKMζ) to maintain synaptic plasticity and memory despite tau-mediated pathogenesis. Thus, our results distinguished KIBRA both as a biomarker of synapse dysfunction and as the foundation for a synapse repair mechanism to reverse cognitive impairment in tauopathy.

## Abstract 2

<p>KIBRA levels in human brain and CSF correlate with tauopathy-related cognitive impairment and the KIBRA C-terminus restores plasticity and memory in a tauopathy mouse model.</p>

## Graphical abstract

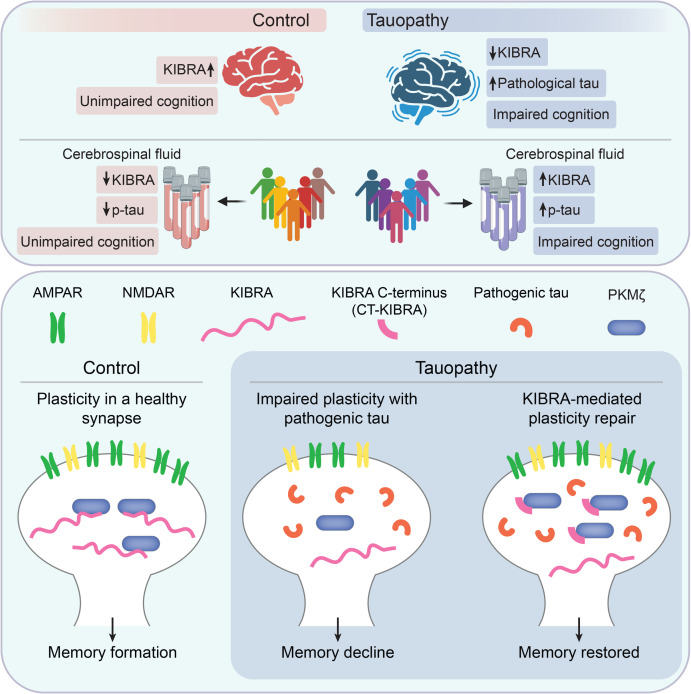

## Introduction

Pathological tau protein accumulates and forms aggregates in the brain in neurodegenerative diseases classified as tauopathies, including Alzheimer’s disease (AD), Pick’s disease, corticobasal degeneration (CBD) and progressive supranuclear palsy (PSP). Aberrant tau posttranslational modifications (PTMs) found in tauopathy brains can alter tau protein function, promote tau aggregation, and trigger toxicity in cells (1, 2). The extent of pathologically modified tau that accumulates in the human brain correlates with the severity of dementia in disease (3, 4). Tau is predominantly localized within axons of healthy neurons, whereas an abundance of pathological tau with PTMs is found at synapses in the AD brain (5–7). Synapse dysfunction is one of the earliest pathophysiological changes in tauopathy mouse models that precedes neurodegeneration and coincides with the start of cognitive impairments (8–10). Long-term potentiation (LTP) is an important plasticity mechanism at synapses in the hippocampus, which underlies the formation of new memories, and LTP is inhibited by tau with mutations that cause familial frontotemporal dementia(FTD) (8, 10, 11), as well as several AD-associated pathogenic forms of tau including hyperacetylated tau (9), hyperphosphorylated tau (12, 13), and tau oligomers (14, 15). Synapses, and their ability to express LTP, are particularly vulnerable to tau-induced toxicity in the brain.

KIdney/BRAin (KIBRA) is a postsynaptic protein encoded by the *WWC1* gene that has a single nucleotide polymorphism linked to memory and risk of late-onset AD in humans (16–19). The KIBRA protein is required for hippocampal LTP and memory in mice (20), and it contains multiple functional domains acting as a postsynaptic scaffold with approximately twenty identified binding partners (21, 22), supporting a critical role for KIBRA in modulating synaptic signaling and strength. In humans, KIBRA protein is significantly diminished in the brain tissue of individuals with severe AD dementia and reduced KIBRA levels are associated with abnormal hyperacetylated tau (9). Moreover, a mimic of hyperacetylated human tau expressed in transgenic mice (tauKQ^high^ mice) obstructs LTP by reducing KIBRA levels at synapses (9), supporting that the loss of KIBRA function at synapses underlies AD-related plasticity and memory impairments.

Here, we quantified cerebrospinal fluid (CSF) KIBRA levels in humans and show that elevated KIBRA in CSF and reduced KIBRA levels in the human brain correlate with pathogenic tau levels and dementia severity in adults with tauopathy. To investigate how KIBRA-mediated signaling modulates synapses in neurons with pathogenic tau, we generated a truncated version of the KIBRA protein, comprised of the C-terminal domain (CT-KIBRA). We found that expression of CT-KIBRA in neurons of transgenic mice with pathogenic tau is sufficient to reverse synaptic plasticity and memory impairments associated with AD and related dementias. Together with the observation that tau-related pathology persists in the mouse brain, this provides evidence that CT-KIBRA promotes synaptic and cognitive resilience to tau toxicity. We further investigated the mechanistic effect of CT-KIBRA on LTP in neurons with pathogenic tau, finding that CT-KIBRA repairs synapse function through its interaction with protein kinase Mζ (PKMζ), an atypical protein kinase C (PKC) isoform that regulates postsynaptic AMPA-type glutamate receptors (AMPARs) and sustains the maintenance of LTP and memory (23–25). These results highlight the potential for a therapeutic strategy to reestablish KIBRA function in neurons, repair synapses, and reverse memory loss in patients with tauopathy.

## Results

### KIBRA levels in human brain and CSF are associated with pathological tau and cognitive impairment in older adults.

**Figure 1:**
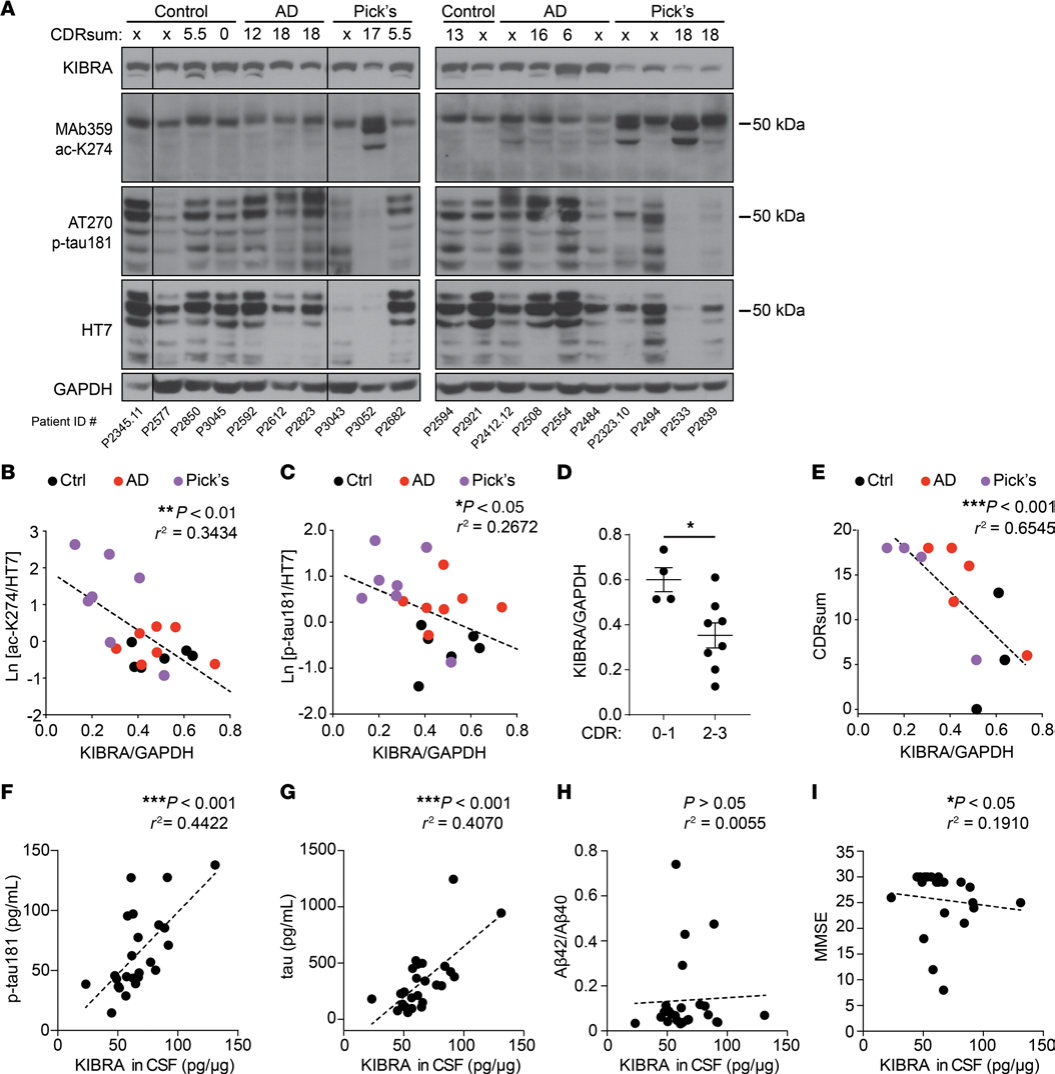
Reduced KIBRA levels in the human brain and increased KIBRA levels in human CSF correlate with pathological tau levels and cognitive impairment in tauopathy. (**A**) Immunoblots of homogenates from the middle temporal gyrus region of human brain classified as control, AD, and Pick’s disease cases based on neuropathological diagnosis. Of the 20 cases analyzed, 12 were assigned a CDRsum score based on cognitive function that are labeled above each lane. Lanes run on the same gel that were not adjacent are denoted by boundary lines. See also Supplemental Table 1. (**B** and **C**) Correlation of KIBRA levels and the amount of soluble tau protein in human brains that is (**B**) acetylated on lysine 274 (ac-K274, MAb359 antibody), and (**C**) phosphorylated on threonine 181 (p-tau181, AT270 antibody). Immunoreactivity of acetylated and phosphorylated tau was quantified relative to total tau levels (HT7) measured from MAb359 and AT270 immunoblots, respectively (*n* = 6–7 cases/group; Spearman’s correlation). (**D**) KIBRA levels quantified in brain homogenate from 12 cases assigned CDR scores (8 of the 20 brain samples used for this study were not assigned CDR scores). Cases with CDR global scores of 0–1, with none-to-mild dementia, were compared to CDR 2–3 cases, with moderate-to-severe dementia (*n* = 4–8 cases/group; *P < 0.05, unpaired Student’s *t* test). Values are given as means ± SEM. (**E**) Relationship between CDRsum score of neuropathological control, AD, and Pick’s disease cases and levels of KIBRA in brain homogenate from the 12 cases assigned CDR scores (*n* = 3–5 cases/group; Spearman’s correlation). (**F**–**H**) Spearman’s correlation analyses show the relationship between KIBRA levels and (**F**) p-tau181, (**G**) total tau, and (**H**) the Aβ42/Aβ40 ratio detected in individual CSF samples (*n* = 24–25 individuals). (**I**) Higher CSF KIBRA levels are associated with worse cognition evaluated by MMSE (*n* = 21 individuals, Spearman’s correlation). See also Supplemental Figure 1 and Supplemental Table 2.

To examine the relationship between KIBRA and pathogenic tau in different tauopathies, we performed immunoblot analyses on human brain homogenates from the middle temporal gyrus of control, AD, and Pick’s disease cases characterized by neuropathology (Figure 1A and Supplemental Table 1; supplemental material available online with this article; https://doi.org/10.1172/JCI169064DS1). Soluble tau levels, detected using an HT7 antibody, were lower in some of the tauopathy cases, which may be due to the redistribution of tau into insoluble aggregates in the brain (26–28). To assess aberrantly acetylated soluble tau in the brains, we used the MAb359 antibody, which recognizes tau acetylated on K274 and labels pathological tau in most tauopathies, including both AD and Pick’s disease (9, 29). The elevated levels of K274 acetylation on soluble tau exhibited a strong relationship with lower KIBRA levels in the brain among the tauopathy cases (Figure 1B). Reduced KIBRA levels also correlated with increased phosphorylation of soluble tau (p-tau181) assayed by the AT270 immunoreactivity (Figure 1C), indicating that KIBRA downregulation is linked to both hyperacetylation and hyperphosphorylation of tau in the brain.

KIBRA levels are significantly diminished in the brain in adults with severe AD compared with people without dementia in the control group (9). Here we explored the relationship between cognition and KIBRA in the brain across stages of dementia in tauopathy. Global Clinical Dementia Rating (CDR) scores were analyzed in twelve participants who ranged in dementia severity from none-to-mild (CDR 0–1) to moderate-to-severe dementia (CDR 2–3). Consistent with previous findings, KIBRA levels were significantly reduced in individuals with moderate-to-severe dementia (CDR 2–3) compared with those with none-to-mild (CDR 0–1) clinical impairment (Figure 1D). Comparisons were also made using the CDR sum of box (CDRsum) scores, which allows for more continuous quantification of the extent of functional impairment compared with global CDR (30, 31). KIBRA levels strongly correlated with degree of functional severity measured by CDRsum across control, AD, and Pick’s disease brains, with the most impaired individuals having the low¬est levels of KIBRA in the brain (Figure 1E).

Synaptic proteins can be detected in human CSF (32), including SNAP-25 (33) and neurogranin (34, 35), and are showing promise as biomarkers of in vivo synapse function in humans. Previous studies suggest that higher levels of several CSF synaptic proteins correlate with greater clinical dysfunction (36). To establish a method for detection of human KIBRA in CSF, we tested a KIBRA ELISA on lysates from HEK293 cells with or without KIBRA overexpression and we confirmed that the ELISA detected significant differences between KIBRA levels expressed at varying protein levels (Supplemental Figure 1A). We next used the ELISA to quantify the amount of KIBRA in CSF of 10 individuals with cognitive impairments due to AD, as well as 15 people in the control group with normal cognition (Supplemental Table 2). KIBRA levels were compared with CSF tau biomarkers including p-tau181 and total tau, which are both increased in the CSF of patients with AD and strongly correlate with worsened cognition (37). CSF KIBRA was significantly increased in individuals with clinically elevated CSF p-tau181 (Supplemental Figure 1B) (38). Furthermore, there was a strong correlation between higher KIBRA levels detected in CSF and increased p-tau181 and total tau levels (Figure 1, F and G). In contrast, KIBRA did not correlate with concentrations of Aβ40, Aβ42 or the ratio of Aβ42/Aβ40 in CSF (Figure 1H and Supplemental Figure 1, C and D), indicating that CSF KIBRA is predominantly associated with biomarkers of tau pathology. To assess CSF KIBRA as a synaptic biomarker related to cognition in AD, we examined the relationship between CSF KIBRA levels and cognitive performances across participants on tasks of global cognition (Mini-Mental State Exam, MMSE). Higher CSF KIBRA levels significantly correlated with lower MMSE (Figure 1I). Together, these results demonstrated that reduced KIBRA in the brain and increased KIBRA in CSF track strongly with clinical disease stage, cognitive impairment, and tau pathology in humans with tauopathy.

### A functional domain of the KIBRA protein reverses tauopathy-related synapse dysfunction.

**Figure 2:**
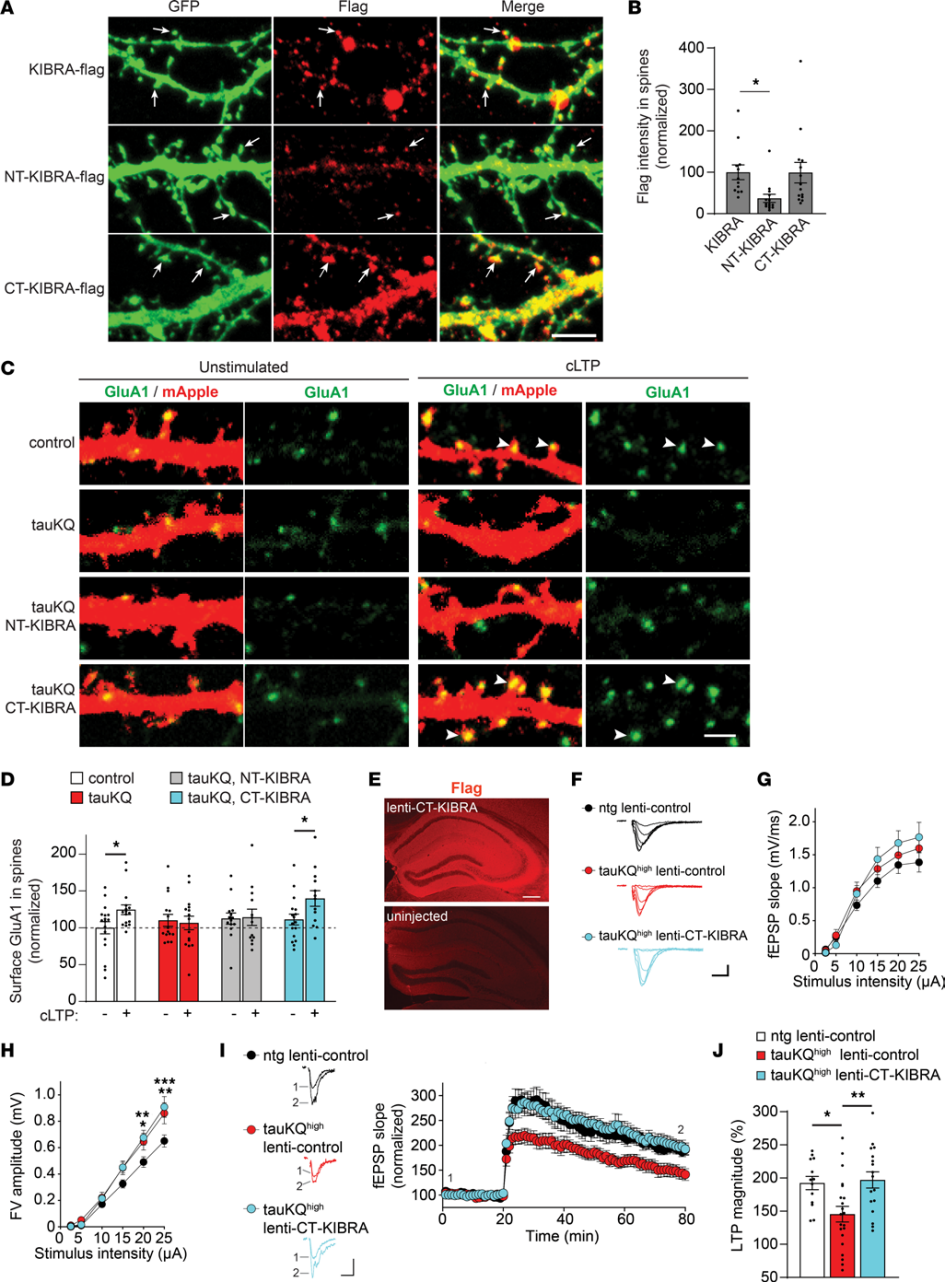
CT-KIBRA reverses the impairment of activity-dependent postsynaptic AMPAR trafficking and LTP caused by pathogenic tau. (**A**) Flag-tagged KIBRA constructs (red) were coexpressed with GFP (green) in cultured rat hippocampal neurons. Full-length KIBRA, NT-KIBRA, and CT-KIBRA were detected in dendritic spines (arrows). Scale bar: 5 μm. See also Supplemental Figure 2. (**B**) Quantification of flag immunoreactivity in dendritic spines of neurons expressing flag-tagged KIBRA, NT-KIBRA, or CT-KIBRA (*n* = 12–14 neurons/group; *P < 0.05, 1-way ANOVA, Bonferroni post hoc analysis versus full-length KIBRA group). (**C**) Images of surface AMPARs (green), labeled with an N-terminal GluA1 antibody, in neurons expressing mApple (red). Neurons were either unstimulated or had chemical LTP (cLTP) induction which enhanced postsynaptic AMPARs in control and CT-KIBRA-expressing tauKQ neurons (arrowheads). Scale bar: 2 μm. (**D**) Surface GluA1 immunofluorescence intensity was quantified in spines (*n* = 13–17 neurons/group; *P < 0.05, unpaired Student’s *t* test). See also Supplemental Figure 2. (**E**) Images of flag immunolabeling (red) detected in the ipsilateral hippocampus of a mouse injected with flag-tagged CT-KIBRA lentivirus and the contralateral side that was not injected with lentivirus. Scale bar: 200 μm. (**F**–**H**) Field recordings were performed in dentate gyrus molecular layer of brain slices from 17–18-month-old mice. (**F**) Field excitatory postsynaptic potential (fEPSP) slopes and fiber volley (FV) amplitudes were measured in response to increasing stimulus intensities applied to perforant pathway inputs. Graphs of mean fEPSP slope (**G**) and FV amplitude (**H**) show that tauKQ^high^ brain slices exhibited increased FV amplitude at the highest stimulus intensities (*n* = 12–14 slices from 4 mice/group; 20 μA stimulus: ntg lenti-control versus taukQ^high^ lenti-control *P < 0.05, ntg lenti-control versus taukQ^high^ lenti-CT-KIBRA **P < 0.01; 25 μA stimulus: ntg lenti-control versus taukQ^high^ lenti-control **P < 0.01, ntg lenti-control versus taukQ^high^ lenti-CT-KIBRA ***P < 0.001, repeated measures 2-way ANOVA, Bonferroni post hoc analyses). Scale bars: 0.5 mV and 5 ms. (**I**) Quantification of the fEPSP slopes 20 minutes before and 60 minutes after TBS to induce LTP was normalized to the average fEPSP slope during the first 20 minutes of the baseline recording for each slice (*n* = 13–21 slices from 5–7 mice/group). Scale bars: 0.4 mV and 10 ms. (**J**) Mean fEPSP slopes at 55–60 minutes after TBS induction representing the magnitude of LTP (*n* = 13–21 slices from 5–7 mice/group; *P < 0.05, **P < 0.01, 1-way ANOVA, Bonferroni post hoc analyses). Values are given as means ± SEM.

We next explored how KIBRA modulates the synaptic pathophysiology caused by pathogenic tau. To dissect the mechanistic effect of the KIBRA protein on tau-mediated synapse dysfunction, we generated constructs to express flag-tagged full-length human KIBRA or truncated forms of the KIBRA protein in neurons (Supplemental Figure 2A). Two functional domains of the KIBRA protein were tested including the 86 residues of the N-terminus containing the WW domains (NT-KIBRA) that interacts with synaptopodin and dendrin (39, 40), and the 187 residues of CT-KIBRA that interacts with members of the PKC family including PKMζ (41–43). The constructs were expressed in cultured rat hippocampal neurons, and immunostaining revealed that full-length human KIBRA and CT-KIBRA localized in dendritic spines, whereas the levels of NT-KIBRA in spines were significantly lower (Figure 2, A and B). We next examined the effect of the distinct KIBRA functional domains on the synaptic plasticity impairment caused by pathogenic tau. Neurons were transfected with either of the truncated KIBRA constructs together with human tau carrying lysine to glutamine mutations at lysine-274 and lysine-281 (tauKQ) to mimic hyperacetylated pathogenic tau that accumulates in the brain in patients with AD and most other tauopathies (9, 29). A chemical induction method, involving glycine treatment in the absence of magnesium, was applied to the neurons to induce LTP, which potentiates synapse function through recruitment of AMPARs to the surface of postsynaptic spines (44, 45). There was a significant increase in the surface immunolabeling of GluA1-containing AMPARs in dendritic spines of control neurons after chemical LTP induction, whereas AMPAR insertion was obstructed in neurons expressing tauKQ with or without NT-KIBRA (Figure 2, C and D). Coexpression of CT-KIBRA with tauKQ in neurons rescued the increase in AMPARs on the spine surface triggered by chemical LTP (Figure 2, C and D). Likewise, the activity-induced recruitment of AMPARs to spines was blocked in neurons expressing human tau carrying the P301L mutation that causes familial FTD, and coexpression of CT-KIBRA with the FTD mutant tau rescued AMPAR recruitment during chemical LTP (Supplemental Figure 2, B and C).

Based on our finding that CT-KIBRA was sufficient to modulate AMPARs at synapses during plasticity, we next examined the effect of CT-KIBRA on LTP in aged transgenic mice expressing high levels of tauKQ (tauKQ^high^). TauKQ^high^ mice have impaired LTP in the dentate gyrus by 6 months of age (9). We generated lentivirus for flag-tagged CT-KIBRA expression in the mouse hippocampus following stereotaxic injection (Figure 2E). To determine the efficacy of CT-KIBRA in reversing synapse dysfunction in aged mice, the CT-KIBRA lentivirus (lenti-CT-KIBRA) or a control lentivirus (lenti-control) was bilaterally injected into the hippocampus of 15–16-month-old tauKQ^high^ mice and nontransgenic (ntg) controls. At 17–18 months of age, field recordings were performed in the dentate gyrus molecular layer with stimulation of the perforant pathway (Figure 2F). Compared with ntg lenti-control mice, dentate granule cells in tauKQ^high^ mice with either lenti-control or lenti-CT-KIBRA exhibited similar basal postsynaptic responses to stimuli, but the fiber volley amplitudes recorded from perforant pathway projections into the dentate gyrus were significantly elevated at the highest stimulus intensities in both control and lenti-CT-KIBRA–treated tauKQ^high^ mice (Figure 2, G and H). This suggests that tauKQ altered the maximum strength of perforant pathway inputs to dentate gyrus in aged mice, however, this presynaptic effect of tauKQ was not modulated by CT-KIBRA. Induction of LTP by theta burst stimulation (TBS) of the perforant pathway triggered a persistent enhancement of the postsynaptic response in ntg lenti-control mice, which was impaired in tauKQ^high^ lenti-control mice (Figure 2I). The magnitude of LTP in dentate granule cells was significantly increased in tauKQ^high^ lenti-CT-KIBRA compared with tauKQ^high^ lenti-control mice, and CT-KIBRA enhanced the persistent strengthening of synapses in tauKQ^high^ mice to the same level as ntg lenti-control mice (Figure 2J). These results support that the C-terminal domain of the KIBRA protein is sufficient to restore AMPAR trafficking and LTP at synapses in hippocampal neurons with pathogenic tau.

### CT-KIBRA improves hippocampus-dependent memory in mice with pathogenic tau, despite tauopathy-related pathology.

**Figure 3:**
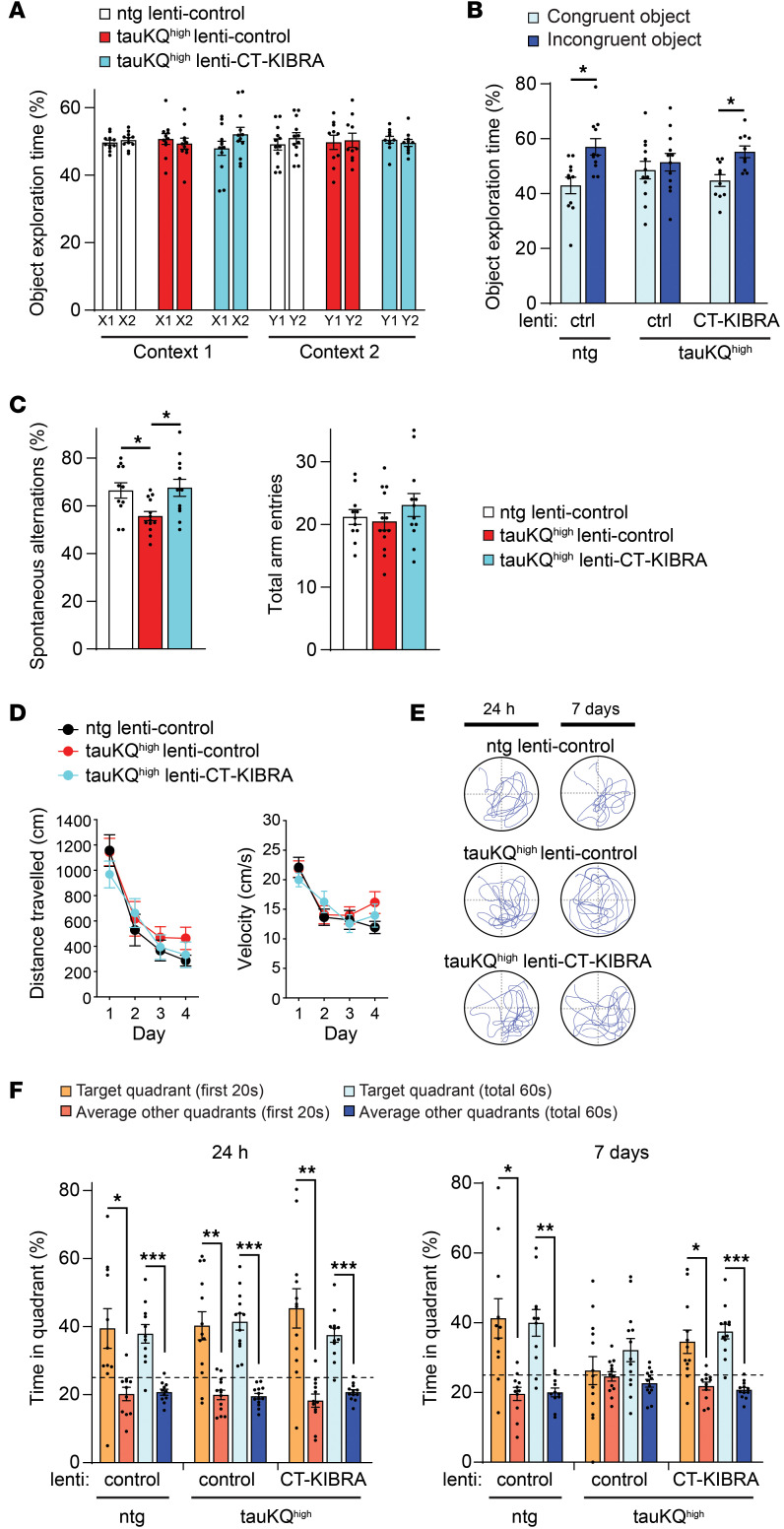
CT-KIBRA expression in hippocampus reverses memory impairment in tauKQ^high^ mice. (**A** and **B**) The object-context discrimination test was used to assess pattern separation memory (*n* = 10–12 mice/group). (**A**) During the sample phase, the mean proportion of time that mice spent exploring 2 identical objects in each context was calculated (X1 and X2 in Context 1, or Y1 and Y2 in Context 2). (**B**) Mean percent time spent exploring the incongruent and congruent objects in both contexts for each group was analyzed during the test phase of the object-context discrimination test (*P < 0.05, paired Student’s *t* test). (**C**) Graphs of the mean percentage of spontaneous alternations completed by the mice in the Y-maze within 5 minutes (left) and the average number of total Y-maze arm entries made by the mice (right) (*n* = 11–13 mice/group; *P < 0.05, 1-way ANOVA, Bonferonni post hoc analyses). (**D**–**F**) Mice were tested for spatial learning and memory in the MWM (*n* = 11–13 mice/group). (**D**) The mean distance traveled to the hidden platform and the average swim velocity were measured during hidden platform training (*P* > 0.05, repeated measures 2-way ANOVA). (**E**) Representative swim paths of mice during the probe trials for spatial memory testing performed at 24 hours and 7 days after hidden platform training. (**F**) Graphs of the mean percent time spent in the target quadrant compared with the average time spent in the other quadrants in the 24 hour (left) and 7-day (right) probe trials. Comparisons between target quadrant and other quadrants were analyzed for both the first 20 second and the total 60 second period of the probe trial for all mice (*P < 0.05, **P < 0.01, and ***P < 0.001, paired Student’s *t* test). The dotted line marks the 25% chance level. Values are given as means ± SEM.

We next assessed the functional impact of CT-KIBRA on hippocampus-dependent memory impairment caused by pathogenic tau in aged mice. TauKQ^high^ mice have pattern separation memory loss by 6–7 months of age coinciding with the LTP deficit in dentate gyrus (9). To test the impact of CT-KIBRA treatment after the onset of memory impairment in aged tauKQ^high^ mice, injections of lenti-CT-KIBRA or lenti-control into bilateral hippocampi were performed on tauKQ^high^ mice and ntg littermates at 10–12 months old and behavior tests were performed 4–6 weeks later. During the sample phase of the object-context discrimination test of pattern separation memory, the mice spent equivalent time exploring the distinct pairs of identical objects in 2 different but similar contexts (Figure 3A). In the memory test phase, 1 of the objects in each pair was switched between the contexts, thus presenting the mice with a familiar object that is incongruent with the context. The ntg mice explored the incongruent object significantly longer than the congruent object (Figure 3B), which is an indication of hippocampus-dependent pattern separation memory. The tauKQ^high^ lenti-control mice demonstrated impaired pattern separation memory, however lenti-CT-KIBRA restored the performance of tauKQ^high^ mice in discrimination of the mismatched object (Figure 3B).

The tauKQ^high^ mice were next tested in the Y-maze to determine whether CT-KIBRA has an impact on hippocampus-dependent working memory. TauKQ^high^ lenti-control mice demonstrated impaired working memory, whereas the proportion of spontaneous alternations completed by tauKQ^high^ lenti-CT-KIBRA mice was significantly increased without affecting the total number of arm entries during the test (Figure 3C). Thus, CT-KIBRA improved spatial working memory in tauKQ^high^ mice. In the Morris water maze (MWM) test of spatial learning and memory, ntg lenti-control, taukQ^high^ lenti-control, and tauKQ^high^ lenti-CT-KIBRA mice showed comparable swim speeds and spatial learning to find the hidden platform during training (Figure 3D). The ntg lenti-control, taukQ^high^ lenti-control, and tauKQ^high^ lenti-CT-KIBRA mice spent a greater fraction of time in the target quadrant in the probe trial 24 hours after the hidden platform training was completed (Figure 3, E and F). Only the ntg lenti-control and tauKQ^high^ lenti-CT-KIBRA mice maintained a significant preference for the target quadrant probe trial after 7 days, whereas tauKQ^high^ lenti-control mice did not (Figure 3, E and F), indicating that CT-KIBRA reversed the long-term spatial memory impairment in tauKQ^high^ mice. Overall, these results support that CT-KIBRA enhances multiple forms of hippocampus-dependent memory that are compromised by pathogenic tau.

**Figure 4:**
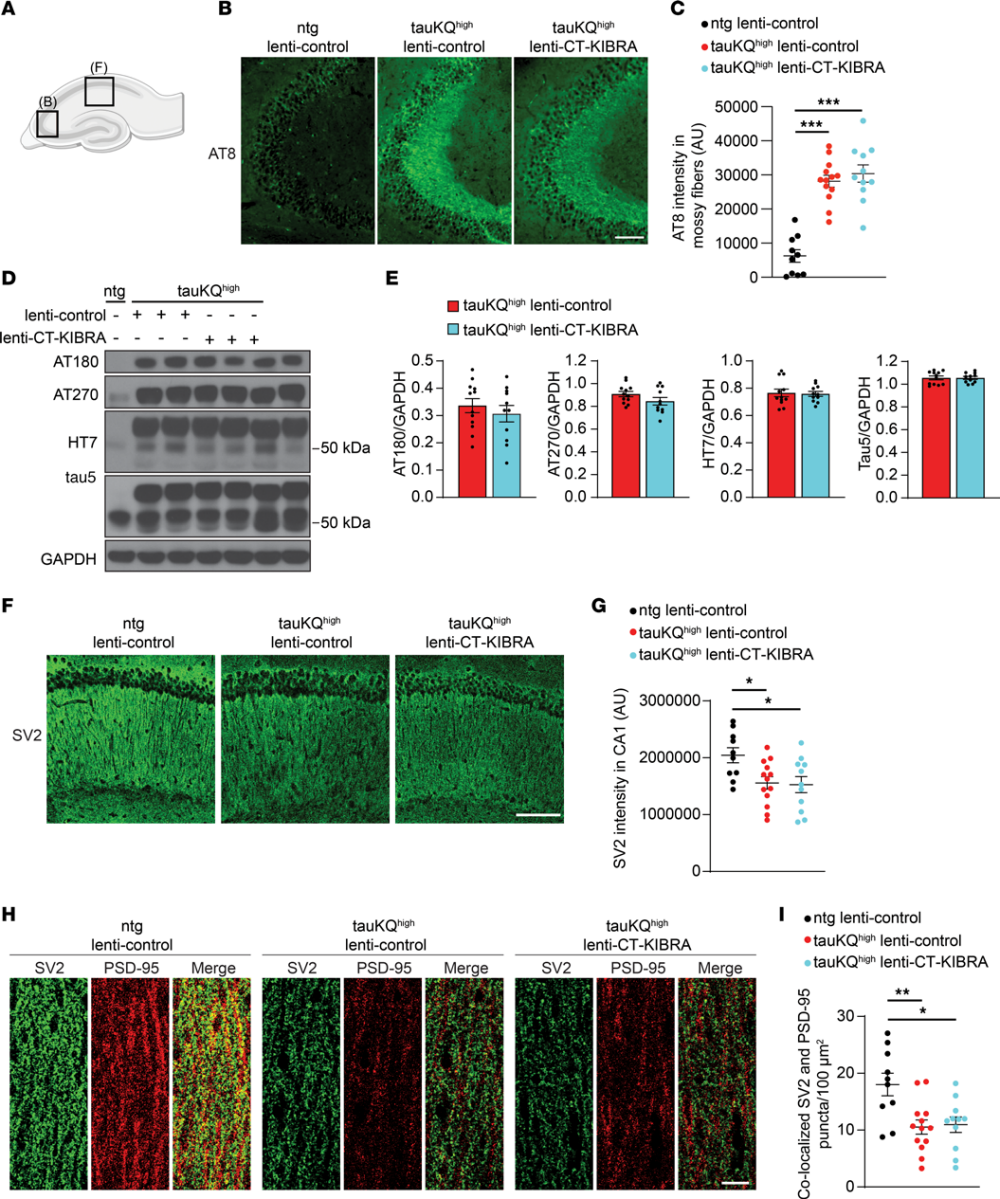
Elevated pathological tau and CA1 synapse loss persist in hippocampus of tauKQ^high^ mice with CT-KIBRA. (**A**) Illustration of a mouse hippocampus depicting where the imaging of mossy fibers (**B**) and CA1 synapses (**F**) was performed. (**B**) Representative images of AT8 immunostaining in the mossy fibers inputs to CA3 in 13–15-month-old tauKQ^high^ mice with or without CT-KIBRA. Scale bar: 100 μm. (**C**) Graph of the mean integrated intensity of AT8 immunofluorescence in the mossy fibers of CA3 (*n* = 10–13 mice/group, ***P < 0.001, 1-way ANOVA, Bonferonni post hoc analyses). (**D**) Representative immunoblots of phosphorylated tau (AT180 and AT270), human tau (HT7), and total tau (Tau5) from hippocampal homogenates of 13–15-month-old ntg lenti-control, tauKQ^high^ lenti-control,and tauKQ^high^ lenti-CT-KIBRA mice. (**E**) Quantification of phosphorylated tau and total tau in hippocampal homogenates normalized to GAPDH (*n* = 10–13 mice/group, *P* > 0.05, unpaired Student’s *t* test). (**F**) Representative confocal images of SV2 immunolabeling as a marker of CA1 synapses. Scale bar: 100 μm. (**G**) Quantification of the mean integrated intensity of SV2 immunofluorescence in CA1 stratum radiatum in 13–15-month-old mice (*n* = 10–13 mice/group; *P < 0.01, 1-way ANOVA, Bonferonni post hoc analyses). Values are given as means ± SEM. See also Supplemental Figure 3 and Supplemental Table 3. (**H**) Representative confocal images of immunostaining of SV2 (green), a presynaptic marker, and PSD-95 (red), a postsynaptic marker, in CA1 stratum radiatum. Scale bar: 10 μm. (**I**) Quantification of colocalized SV2 and PSD-95 puncta density in CA1 stratum radiatum (*n* = 10–13 mice/group; *P < 0.05, **P < 0.01, 1-way ANOVA, Bonferonni post hoc analyses).

We next examined whether CT-KIBRA mitigated pathology in the brain caused by expression of the acetylated tau mimic. CT-KIBRA did not prevent the accumulation of tau phosphorylated at serine 202/205 (AT8) that was significantly increased in mossy fibers in the hippocampus of 13–15-month-old tauKQ^high^ mice (Figure 4, A–C). Total levels of human tau and tau phosphorylation at threonine 231 (AT180) and threonine 181 (AT270) in the hippocampus of tauKQ^high^ mice were also not altered by CT-KIBRA expression (Figure 4, D and E). Synaptic vesicle associated protein 2 (SV2) has been used as a marker of synapse loss in human AD brain (46, 47). To assess synapse deterioration in tauKQ^high^ mice, we analyzed immunolabeling of SV2 and found that it was significantly decreased in the CA1 region of the hippocampus in tauKQ^high^ with or without CT-KIBRA compared with ntg controls (Figure 4, F and G). Moreover, the colocalization of presynaptic SV2 and postsynaptic PSD-95 immunolabeling was markedly reduced in CA1 stratum radiatum of tauKQ^high^ mice with and without CT-KIBRA (Figure 4, H and I). Thus, while CT-KIBRA was sufficient to restore hippocampal synaptic plasticity, it was not sufficient to reverse tau-mediated synapse loss in the CA1 region. SV2 immunolabeling in the molecular layer of the dentate gyrus was not altered in tauKQ-expressing mice compared with controls (Supplemental Figure 3, A and B), indicating CA1 region–specific synapse loss in the hippocampus. Mass spectrometry analysis of protein extracted from whole hippocampus of tauKQ^high^ lenti-control and tauKQ^high^ lenti-CT-KIBRA mice was performed to monitor changes in the global proteome compared with ntg lenti-control mice (Supplemental Figure 3C and Supplemental Table 3). Notably, of the 64 hits identified that were either significantly increased or decreased in tauKQ^high^ compared with ntg mice, 33% of them changed in the hippocampi of both tauKQ^high^ lenti-control and tauKQ^high^ lenti-CT-KIBRA mice. A comparison of tauKQ^high^ mice with or without CT-KIBRA also yielded no significant differences (Q < 0.05) in the abundance of the proteins that were identified (Supplemental Table 3). Proteins that were upregulated in tauKQ^high^ lenti-control and tauKQ^high^ lenti-CT-KIBRA mice compared with ntg lenti-control mice included microtubule and cytoskeleton components as well as axonal proteins (Supplemental Figure 3D and Supplemental Table 3). Specific synaptic proteins, including Synapsin-1 and SynGAP, were significantly reduced in whole hippocampus lysates from both tauKQ^high^ lenti-control and from tauKQ^high^ lenti-CT-KIBRA mice compared with ntg lenti-control mice, whereas total expression of glutamate receptor subunits remained unaffected (Supplemental Figure 3, E–H, and Supplemental Table 3). The density of neurons, assessed by NeuN immunolabeling, in CA1 and dentate gyrus was not significantly changed in tauKQ^high^ mice with or without CT-KIBRA expression compared with ntg lenti-control mice (Supplemental Figure 3, I and J), suggesting that neuron loss did not contribute to synaptic pathophysiology in tauKQ^high^ mice. Together, these findings suggest that CT-KIBRA restores synaptic plasticity and reverses tau-induced memory impairment without altering levels of pathological tau in the brain and without recovering the CA1 synapses lost in the hippocampus.

### CT-KIBRA stabilizes and enhances PKMζ at synapses with pathogenic tau.

**Figure 5:**
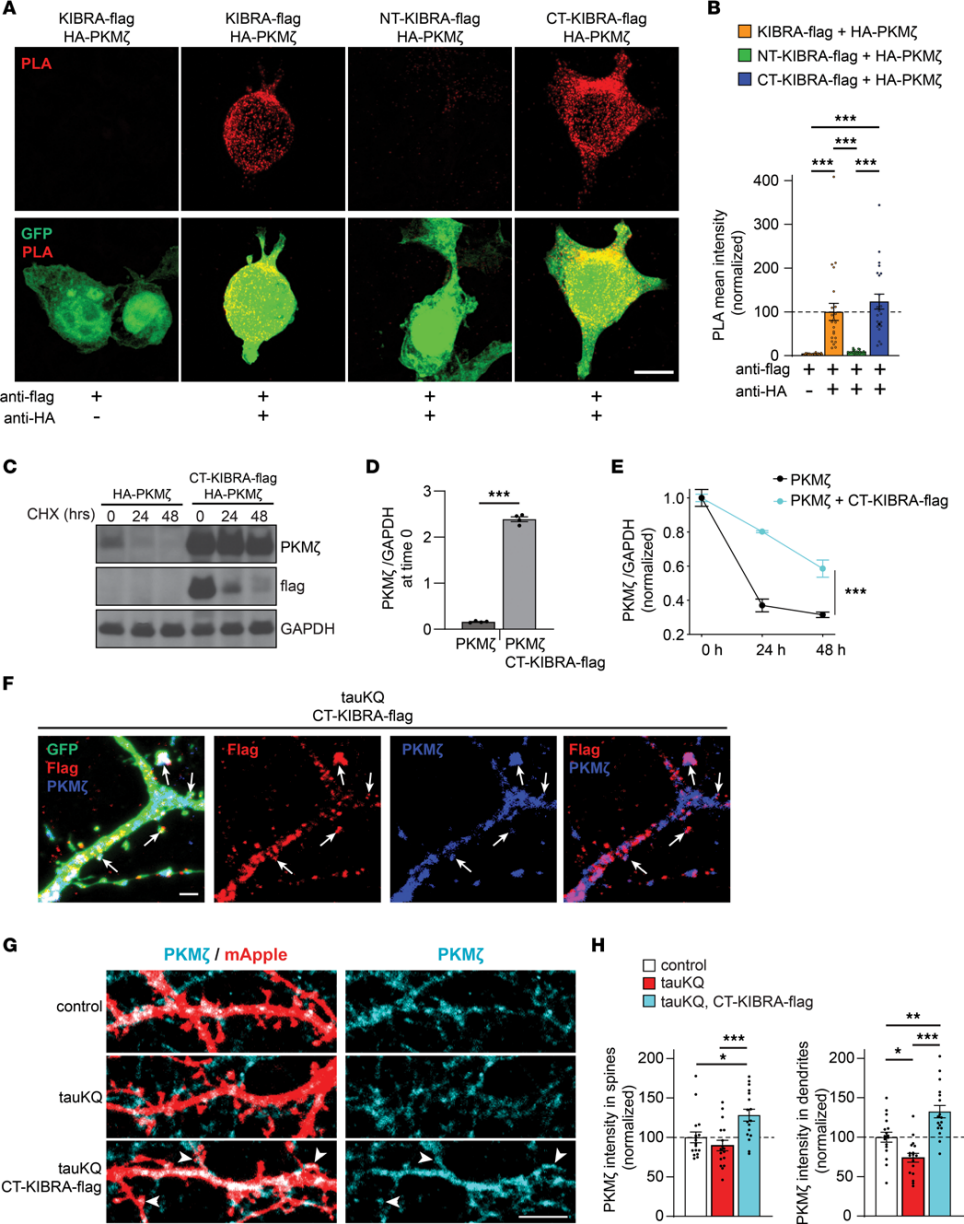
CT-KIBRA interacts with and stabilizes PKMζ at synapses in neurons with pathogenic tau. (**A**) Images of HEK293 cells coexpressing GFP (green) with HA-PKMζ and flag-tagged KIBRA variants. The PLA was applied to HEK293 cells using anti-HA and anti-flag antibodies to label only the interacting PKMζ and KIBRA variants (red). Scale bar: 10 μm. (**B**) Graph of mean PLA intensity quantification from HEK293 cells coexpressing HA-PKMζ with flag-tagged KIBRA variants normalized to PLA intensity of cells with full-length KIBRA expression (*n* = 15–20 cells/group; ***P < 0.001, 1-way ANOVA, Bonferonni post hoc analyses). See also Supplemental Figure 4. (**C**–**E**) HEK293 cells expressing HA-PKMζ with or without CT-KIBRA-flag were treated with CHX to monitor PKMζ degradation for 24 hours or 48 hours. (**C**) Immunoblots from HEK293 cells treated with CHX. (**D**) Quantification of PKMζ levels normalized to GAPDH at time 0 before CHX treatment (*n* = 4 replicates/group; ***P < 0.001, unpaired Student’s *t* test). (**E**) Graph showing HA-PKMζ stability in HEK293 cells with or without CT-KIBRA-flag. HA-PKMζ immunoreactivity was normalized to basal levels at time 0 (*n* = 4 replicates/group; ***P < 0.001, 2-way ANOVA, Bonferonni post hoc analyses). See also Supplemental Figure 5. (**F**) Coimmunolabeling of endogenous PKMζ (blue) and CT-KIBRA-flag (red) in cultured hippocampal neurons with GFP (green) and tauKQ. CT-KIBRA colocalized with PKMζ in postsynaptic spines (arrows) and within dendrites. Scale bar: 2 μm. (**G**) Images of PKMζ immunolabeling (cyan) in dendrites from mApple-expressing (red) cultured hippocampal neurons with or without tauKQ and CT-KIBRA expression. PKMζ immunoreactivity was assessed in spines (arrowheads). Scale bar: 5 μm. (**H**) Graph of mean PKMζ integrated intensity quantified in postsynaptic spines and dendrites normalized to control neurons without tauKQ expression (*n* = 17 cells/group; *P < 0.05, ***P < 0.001, 1-way ANOVA, Bonferonni post hoc analyses). Values are given as means ± SEM. See also Supplemental Figure 5.

Previous studies established that KIBRA binds to PKMζ and prevents PKMζ degradation (41, 48). KIBRA also interacts with PICK1, which modulates AMPAR trafficking during plasticity (20). To examine the interaction of CT-KIBRA with PKMζ and PICK1, we used a proximity ligation assay (PLA), which detects protein-protein interactors within close proximity (under 40 nm) (49). When expressed in HEK293 cells, HA-tagged PKMζ exhibited significantly stronger PLA signal with both full-length KIBRA and CT-KIBRA compared with NT-KIBRA (Figure 5, A and B). These results are consistent with the requirement of the KIBRA C-terminus for PKMζ binding (41) and confirm that the binding affinities of PKMζ to CT-KIBRA and full-length KIBRA are comparable. The PLA signal intensity in HEK293 cells expressing HA-tagged PICK1 together with full-length KIBRA was significantly higher than in cells coexpressing NT-KIBRA or CT-KIBRA (Supplemental Figure 4, A and B), demonstrating that CT-KIBRA has a weaker association with PICK1 than full-length KIBRA. We next tested the effect of CT-KIBRA on PKMζ degradation. When HEK293 cells expressing PKMζ alone were treated with cycloheximide (CHX) to block protein synthesis, PKMζ levels quickly declined over 48 hours (Figure 5, C and E). CT-KIBRA coexpression increased the total amount of PKMζ protein in HEK293 cells (Figure 5, C and D) without changing *PKM*ζ mRNA levels (Supplemental Figure 5, A and B). Consistent with the stabilizing effect of CT-KIBRA on PKMζ, the degradation of PKMζ following CHX treatment was significantly slower in cells with CT-KIBRA compared with those that only expressed PKMζ (Figure 5, C and E).

We next asked whether KIBRA stabilized PKMζ in postsynaptic spines. Flag-tagged CT-KIBRA colocalized with endogenous PKMζ within postsynaptic spines and throughout the dendrites of cultured hippocampal neurons expressing tauKQ (Figure 5F). To monitor the effect of CT-KIBRA on basal postsynaptic PKMζ levels, cultured hippocampal neurons were cotransfected with mApple and tauKQ with or without CT-KIBRA and immunostained for PKMζ. TauKQ neurons had reduced dendritic PKMζ levels compared with control neurons, whereas tauKQ neurons with CT-KIBRA had significantly increased PKMζ levels in both dendrites and postsynaptic spines compared with neurons with tauKQ alone or control neurons (Figure 5, G and H). CT-KIBRA expression also increased PKMζ within postsynaptic spines in control neurons (Supplemental Figure 5, C and D). These data support that the CT-KIBRA and PKMζ interaction stabilizes and enhances postsynaptic PKMζ levels under basal conditions in neurons with pathogenic tau.

### CT-KIBRA protects against PKMζ downregulation associated with plasticity and memory impairments.

**Figure 6:**
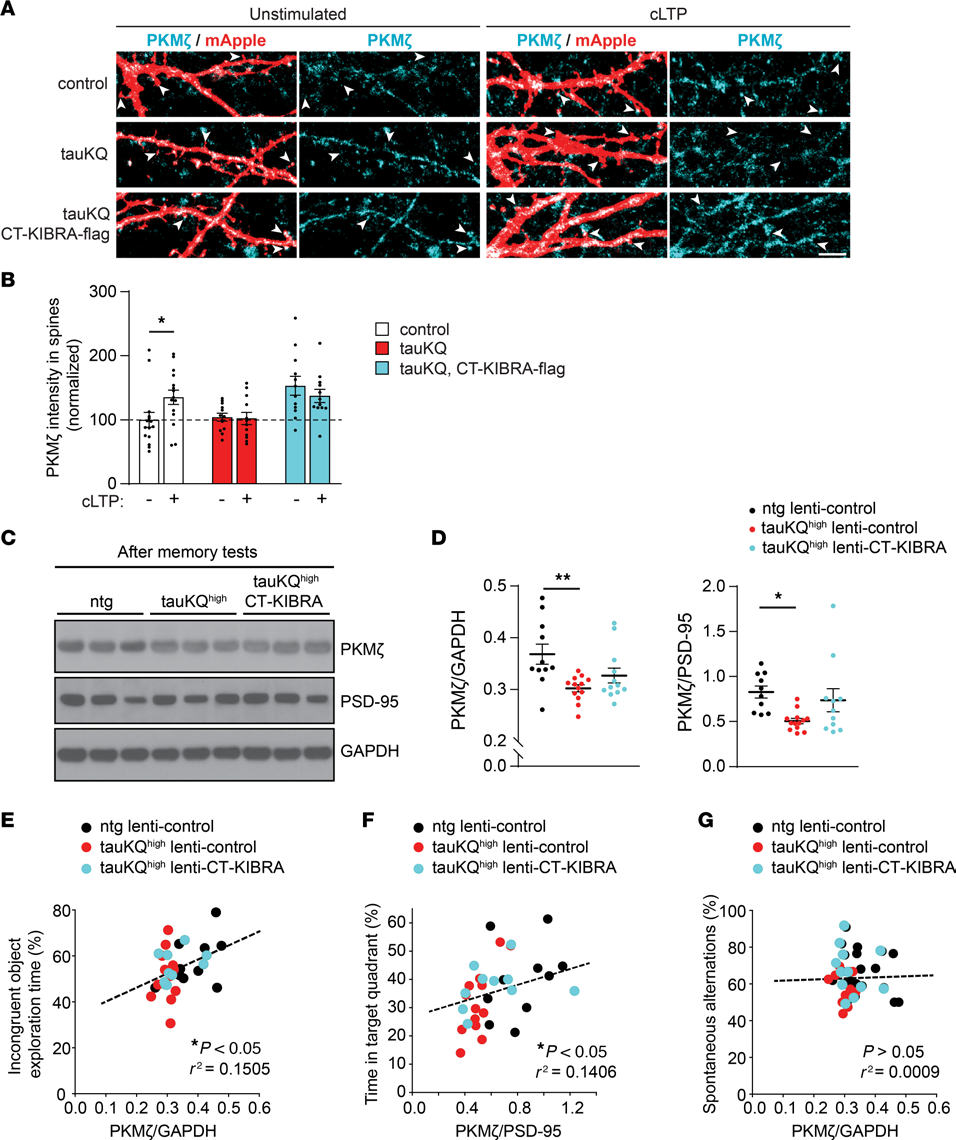
CT-KIBRA maintains plasticity-related PKMζ levels underlying resilience to tau-mediated synaptic and memory deficits. (**A**) Representative images of dendritic spines on cultured hippocampal neurons expressing mApple (red) with or without tauKQ and CT-KIBRA. Immunolabeling of endogenous PKMζ (cyan) was monitored in spines on neurons that were unstimulated or subjected to cLTP treatment (arrowheads). Scale bar: 5 μm. (**B**) Quantification of PKMζ immunoreactivity in postsynaptic spines with or without cLTP induction normalized to postsynaptic PKMζ levels in control unstimulated neurons (*n* = 12–15 cells/group; *P < 0.05, unpaired Student’s *t* test). (**C**) Representative immunoblots of PKMζ, PSD-95, and GAPDH from hippocampal homogenates of 3 individual ntg lenti-control, taukQ^high^ lenti-control and taukQ^high^ lenti-CT-KIBRA mice. (**D**) Quantification of total PKMζ levels in hippocampal homogenates normalized to GAPDH and to PSD-95 (*n* = 10–13 mice/group; *P < 0.05, **P < 0.01, 1-way ANOVA, Bonferonni post hoc analyses). Values are given as means ± SEM. See also Supplemental Figure 6. (**E**) Pearson correlation analyses between PKMζ levels relative to GAPDH analyzed from hippocampal homogenates and the percent time each mouse spent exploring the incongruent object in the object-context discrimination test of pattern separation memory (*n* = 10–12 mice/group). (**F**) Pearson correlation analyses of the proportion of time each mouse spent in the target quadrant of the MWM during the 7-day probe test and the corresponding PKMζ levels in hippocampus relative to PSD-95 (*n* = 10–13 mice/group). (**G**) Pearson correlation analyses of spontaneous alternations made in the Y-maze test and total PKMζ levels in hippocampus (*n* = 11–13 mice/group).

A persistent increase in PKMζ levels occurs in rodent neurons after LTP induction (23, 50, 51). In cultured neurons expressing mApple, we found that PKMζ levels were increased within spines 24 hours after chemical LTP induction compared with neurons that were not stimulated (Figure 6, A and B), in agreement with previous findings in cultured neurons (51). This enhancement of PKMζ was blocked in neurons expressing tauKQ, whereas coexpression of CT-KIBRA increased PKMζ immunostaining in spines under both unstimulated and chemical LTP conditions (Figure 6, A and B). The elevated PKMζ levels after LTP induction were comparable in control neurons and in tauKQ neurons with CT-KIBRA. These findings support that pathogenic acetylated tau blocks the increase in PKMζ following LTP induction, which can be rescued by CT-KIBRA-mediated stabilization of PKMζ in spines.

Basal PKMζ levels in hippocampus were significantly reduced in untrained 12–14-month-old tauKQ^high^ mice compared with littermate controls, and the downregulation remained following normalization of PKMζ levels to PSD-95 (Supplemental Figure 6, A and B), indicating a specific effect on PKMζ levels relative to another postsynaptic protein. After memory formation in behavioral tests on mice, PKMζ levels are enhanced in the hippocampus (23, 52, 53). We further assessed the impact of tauKQ on PKMζ levels following memory formation. PKMζ was quantified in hippocampal lysates from ntg lenti-control, tauKQ^high^ lenti-control, and tauKQ^high^ lenti-CT-KIBRA mice that completed hippocampus-dependent memory tests (Figure 3). After memory tests, total PKMζ levels in the hippocampus of tauKQ^high^ lenti-control mice remained significantly lower than levels in ntg mice with or without normalization to PSD-95. However, tauKQ^high^ mice with CT-KIBRA showed a recovery of PKMζ close to control levels (Figure 6, C and D), suggesting that CT-KIBRA expression maintained PKMζ levels in hippocampus of tauKQ^high^ mice after memory tests. PICK1 was evaluated in the same mice, but the PICK1 levels were not significantly different in tauKQ^high^ lenti-control mice compared with ntg lenti-control mice (Supplemental Figure 6, C and D).

Experiments using acute PKMζ knockdown in hippocampus suggest that PKMζ plays a role in the maintenance of hippocampus-dependent long-term memory (53, 54), and the magnitude of PKMζ enhancement after learning correlates with the capacity for long-term memory in mice (53). Consistent with these studies, we found a significant correlation between hippocampal PKMζ levels and long-term memory among the ntg lenti-control, tauKQ^high^ lenti-control, and tauKQ^high^ lenti-CT-KIBRA mice. Higher levels of PKMζ were associated with better memory retention in both the object-context discrimination test and the MWM test (Figure 6, E and F). PKMζ levels did not correlate with the performance of the mice in the Y-maze test of short-term working memory (Figure 6G). These findings are consistent with the role of PKMζ in long-term as opposed to short-term memory. Given that we found a recovery of short-term memory in tauKQ^high^ lenti-CT-KIBRA mice that did not correlate with PKMζ levels, CT-KIBRA may also enhance synapse function and other cognitive skills through additional mechanisms not yet identified.

### The CT-KIBRA and PKMζ interaction drives LTP repair in neurons with pathogenic tau.

**Figure 7:**
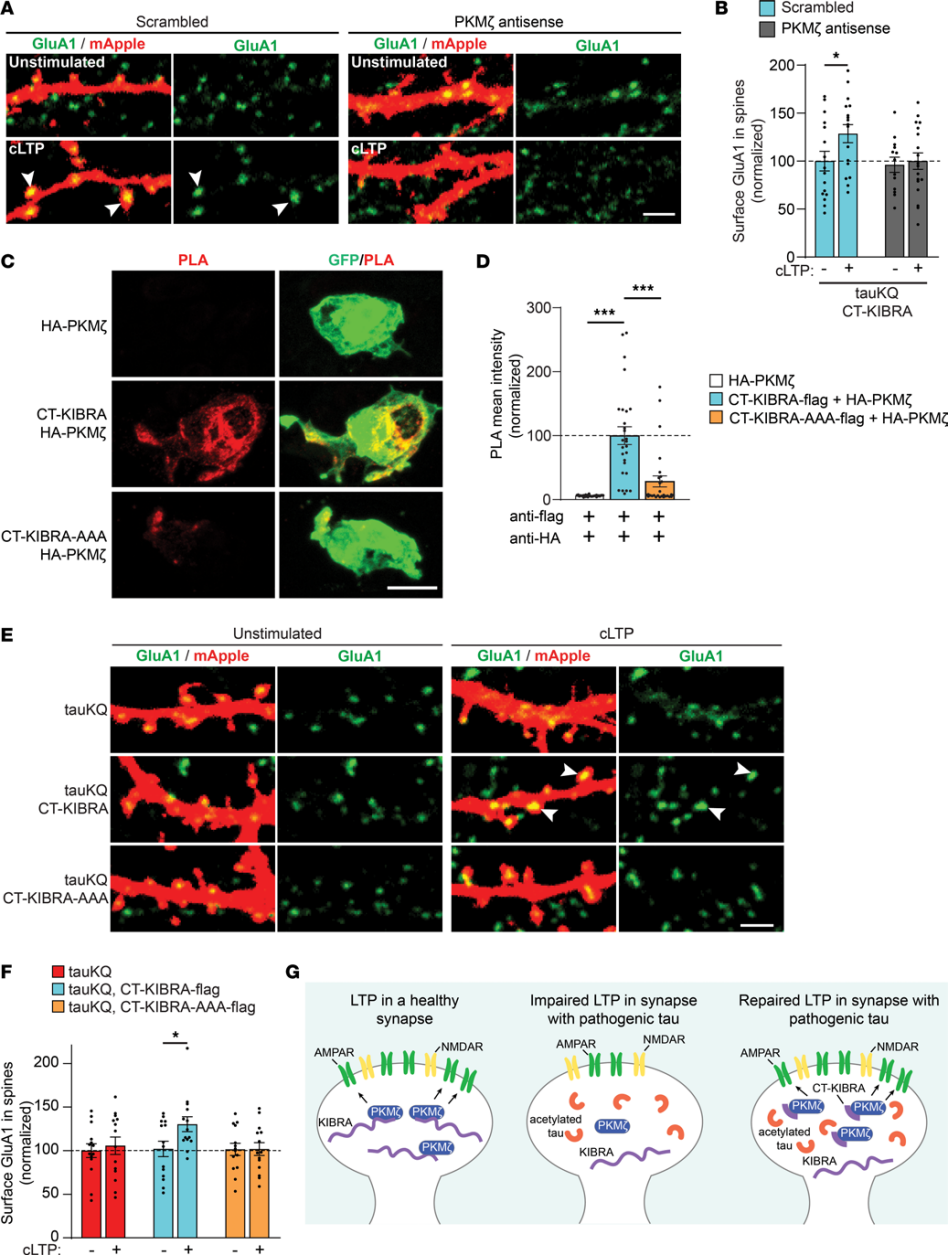
CT-KIBRA restores AMPAR trafficking during LTP by interacting with PKMζ in neurons with pathogenic tau. (**A**) Representative confocal images of surface GluA1 immunostaining (green) in spines of cultured hippocampal neurons expressing mApple (red), tauKQ, and CT-KIBRA. Unstimulated and cLTP neurons were treated with a *PKM*ζ antisense or a scrambled control oligodeoxynucleotide. Scale bar: 2 μm. (**B**) Quantification of the surface GluA1 immunofluorescence in spines normalized to unstimulated neurons treated with the scrambled oligodeoxynucleotide (*n* = 13–18 neurons/group; *P < 0.05, unpaired Student’s *t* test). See also Supplemental Figure 7, A–D. (**C**) Images of HEK293 cells transfected with GFP (green) and HA-PKMζ together with flag-CT-KIBRA or a flag-CT-KIBRA-AAA mutant carrying R965A, S967A, and R969A mutations. Anti-flag and anti-HA antibodies were used for detection of PLA signal (red) signifying the close proximity of flag-CT-KIBRA and HA-PKMζ in the HEK293 cells. Scale bar: 10 μm. (**D**) Quantification of the mean PLA fluorescence intensity detected in HEK293 cells transfected with HA-PKMζ alone (control), or cotransfected with flag-CT-KIBRA constructs (*n* = 27–30 cells/group; ***P < 0.001, 1-way ANOVA, Bonferonni post hoc analyses). (**E**) Representative confocal images of dendrites and spines on neurons cotransfected with mApple (red) and tauKQ with CT-KIBRA or CT-KIBRA-AAA. Scale bar: 2 μm. (**F**) Quantification of surface GluA1 immunofluorescence in spines of tauKQ-expressing neurons showing that cLTP-induced postsynaptic receptor insertion is reestablished by CT-KIBRA, but not CT-KIBRA-AAA. GluA1 levels were normalized to the intensity of staining in spines of unstimulated tauKQ neurons (*n* = 14 neurons/group; *P < 0.05, unpaired Student’s *t* test). Values are given as means ± SEM. See also Supplemental Figure 7, E and F. (**G**) Model depicting the impact of KIBRA and PKMζ on postsynaptic AMPAR trafficking during NMDA receptor–dependent (NMDAR-dependent) LTP in healthy conditions (left) and in tauopathy (middle). Expression of CT-KIBRA in tauopathy neurons (right) with high pathogenic acetylated tau levels can restore postsynaptic AMPAR recruitment during plasticity, which mechanistically involves the interaction between CT-KIBRA and PKMζ.

To investigate the mechanistic relationship between CT-KIBRA and PKMζ in plasticity, we examined whether elevated PKMζ levels are required for CT-KIBRA to restore AMPAR trafficking at synapses during LTP. An antisense oligodeoxynucleotide (*PKM*ζ-antisense) was chosen to acutely block PKMζ expression in neurons to avoid compensation by other PKC isoforms (23), and because the ζ inhibitory peptide (ZIP) is not a specific inhibitor of PKMζ (55, 56). Cultured hippocampal neurons coexpressing tauKQ and CT-KIBRA were treated with *PKM*ζ-antisense or a scrambled control 1 hour before induction and throughout the expression of chemical LTP. The CT-KIBRA–mediated rescue of activity-induced postsynaptic AMPAR recruitment in tauKQ neurons was blocked by *PKM*ζ-antisense (Figure 7, A and B). We then tested whether overexpression of PKMζ in neurons with pathogenic tau was sufficient to rescue synaptic plasticity. HA-tagged PKMζ was expressed in neurons, and immunolabeling with an HA antibody revealed comparable levels of HA-PKMζ localized in spines of neurons with or without tauKQ (Supplemental Figure 7, A and B). Interestingly, the overexpression of HA-tagged PKMζ was not sufficient to restore AMPAR trafficking in tauKQ neurons in the absence of CT-KIBRA (Supplemental Figure 7, C and D), supporting the critical role for KIBRA in the reversal of the tau-mediated plasticity deficit.

Mutations introduced at specific residues within the C-terminal domain of KIBRA block the interaction of KIBRA with PKMζ (41). We took advantage of these mutations to test the role of CT-KIBRA and PKMζ binding in the mechanism underlying the functional repair of synapses with pathogenic tau. We generated a CT-KIBRA-AAA mutant in which 3 residues, R965, S967, and R969, were changed to alanine residues (R965A, S967A, and R969A), which blocks the binding of KIBRA to PKMζ (41). Using the PLA method in HEK293 cells, we confirmed that CT-KIBRA-AAA demonstrated a significantly diminished interaction with HA-PKMζ (Figure 7, C and D). In cultured neurons, the CT-KIBRA-AAA mutant was detected in dendritic spines at similar levels to WT CT-KIBRA (Supplemental Figure 7, E and F). When tested in neurons with chemical LTP, tauKQ consistently impeded postsynaptic AMPAR recruitment during plasticity, but unlike WT CT-KIBRA, the CT-KIBRA-AAA mutant was not sufficient to reverse the impairment (Figure 7, E and F). These findings suggest that CT-KIBRA overcomes the plasticity impairment caused by pathogenic tau by promoting synaptic resilience through its interaction with and modulation of PKMζ (Figure 7G).

## Discussion

Our findings highlight the potential of KIBRA-mediated synapse repair as a therapeutic approach to alleviate memory loss associated with tauopathy. This targeted strategy to restore a precise functional role of KIBRA in synaptic plasticity could be leveraged to counteract the loss of KIBRA in the human brain associated with cognitive impairment in tauopathy. Interestingly, CT-KIBRA improved memory performance and restored LTP in mice despite the accumulation of pathological tau in the brain, supporting that CT-KIBRA rescues mechanisms that promote synaptic resilience to tau toxicity in the brain. The restorative effect of CT-KIBRA involved its binding to and stabilization of PKMζ to promote postsynaptic AMPAR trafficking during plasticity, enabling synapses to maintain function and overcome the adverse effects of pathogenic tau.

Our study provides evidence that CT-KIBRA can improve memory when expressed in the hippocampus after the onset of memory loss caused by pathogenic tau. This supports that CT-KIBRA recovers memory processes by repairing the molecular signaling at synapses required for plasticity. The enhancement of KIBRA signaling may have therapeutic potential broadly across different tauopathies because downregulated KIBRA levels were associated with elevated pathological tau in human brains across AD and Pick’s disease cases. CT-KIBRA improved synaptic plasticity and memory performance in a model of abnormal hyperacetylated tau, which is a common pathology found in most tauopathies, including AD, Pick’s disease, CBD, and PSP (2, 29, 57). CT-KIBRA also restored synaptic plasticity in neurons expressing human P301L tau, confirming the protective effect of CT-KIBRA against tau toxicity associated with FTD. Nonetheless, it will be important to evaluate the impact of CT-KIBRA on synapse dysfunction and cognition in other tauopathy and neurodegenerative disease models. Notably, the mechanisms by which tau drives pathophysiology in neurons in disease are complex, involving not only synapse dysfunction and synapse loss (8–10, 58, 59), but also dysregulation of axonal transport and axon initial segment function, as well as axon degeneration (60–62), altered mitochondrial bioenergetics (63), nuclear transport disruption (64, 65), altered proteostasis (66), and cytoskeletal destabilization (67, 68). Given that CT-KIBRA did not affect tau levels or synapse loss in the hippocampus, it likely plays a specific role in modulating synaptic plasticity.

KIBRA-deficient mice have reduced PKMζ protein levels in the hippocampus and impaired plasticity and memory loss (41, 69), which is consistent with a key role for KIBRA in PKMζ stabilization and the maintenance of synaptic plasticity. Our results show that tauKQ^high^ mice, which have reduced KIBRA at synapses (9), also had decreased PKMζ levels in the hippocampus associated with impaired LTP and hippocampus-dependent memory. Increased forms of pathological tau — including hyperacetylated and hyperphosphorylated tau — in human brain tissue coincided with downregulation of KIBRA, which may be driven by both tau-mediated synapse dysregulation and neuron loss. Aggregates of PKMζ have been observed in neurofibrillary tangles containing hyperphosphorylated tau in the AD brain (70), but whether KIBRA is also bound to PKMζ-containing insoluble aggregates remains to be established. Nevertheless, the prominent correlation between KIBRA downregulation in the human brain and dementia severity suggests that KIBRA functions as a critical synaptic scaffolding protein in plasticity that can impact cognition by modulating PKMζ and other KIBRA-binding proteins at synapses.

The persistent increase in postsynaptic PKMζ levels following plasticity induction underlies the maintenance of LTP (50), and we found that this activity-dependent increase in PKMζ was blocked in neurons with pathogenic tau. Under basal conditions, CT-KIBRA increased postsynaptic PKMζ in tauKQ-expressing neurons above control levels, but following plasticity induction, the enhanced PKMζ levels in control neurons was comparable to the PKMζ levels in tauKQ neurons with CT-KIBRA. This suggests that CT-KIBRA had a protective effect in neurons with pathogenic tau by maintaining the elevated PKMζ levels during synaptic plasticity. Interestingly, CT-KIBRA expression enhanced the PKMζ levels in the spines of tauKQ neurons with or without chemical LTP induction; yet, CT-KIBRA only increased postsynaptic AMPAR trafficking following chemical LTP without altering AMPARs in unstimulated neurons. These findings indicate that CT-KIBRA restores potentiation at synapses both by maintaining elevated PKMζ levels and by enabling an additional activity-dependent signal, which remains to be determined, that drives the AMPAR recruitment after LTP induction. Surprisingly, PKMζ overexpression alone was incapable of rescuing plasticity in tauKQ neurons, supporting that CT-KIBRA has a mechanistic effect during synaptic plasticity beyond enhancing PKMζ levels. Intriguing mechanisms that may be involved in the plasticity repair orchestrated by CT-KIBRA could include the activation of PKMζ or other PKC isoforms, the phosphorylation of the C-terminus of KIBRA by PKMζ (43), or the phosphorylation of AMPARs. CT-KIBRA may also modify synapse strength through binding to other PKC isoforms implicated in synaptic plasticity regulation, such as PKCγ, α, β, and ι, which were reduced in postsynaptic fractions prepared from KIBRA-KO mice (69). While our study highlighted the critical role for KIBRA in modulating synapse repair to recover memory and showed that PKMζ overexpression alone was not sufficient to restore plasticity in neurons with pathogenic tau, a recent study found that the memory impairments caused by oligomeric amyloid β toxicity in rats were alleviated by PKMζ overexpression in the hippocampus (71). Thus, enhanced PKMζ levels may be sufficient to promote synapse function under certain pathogenic conditions but not others. How KIBRA, PKMζ, and other PKC family members could be modulated to restore plasticity in neurodegenerative diseases deserves further investigation.

The KIBRA protein has multiple functional domains that interact with numerous postsynaptic proteins to modulate synapse function; here, we identified a specific KIBRA domain and interaction that can have a beneficial impact in the context of disease. The N-terminal WW domains of KIBRA bind to dendrin, and an inhibitory peptide that blocks this interaction reduced KIBRA levels and AMPARs at synapses on neurons, blocked LTP, and impaired memory in mice (39), indicating that the interaction between KIBRA and dendrin regulates synaptic KIBRA localization and plasticity. We expressed NT-KIBRA, comprising only the WW domains, in neurons and detected it in dendritic spines, although to a lesser degree compared with full-length KIBRA, and it did not rescue LTP in neurons with pathogenic tau. This suggests that, while the dendrin/KIBRA WW domain interaction is necessary for plasticity in healthy neurons, it is not sufficient to reverse the plasticity impairment caused by pathogenic tau. The binding of KIBRA to PICK1 can also modulate activity-dependent AMPAR trafficking in neurons (20). Our findings show a weakened interaction of CT-KIBRA with PICK1 compared with full-length KIBRA and a modest effect of pathogenic tau on PICK1 protein levels. However, whether CT-KIBRA could influence PICK1-dependent AMPAR trafficking in neurons, or additional plasticity mechanisms, remains to be determined. In future studies it will be important to consider that the impact KIBRA has on synapse function and plasticity through its interactions with postsynaptic proteins may depend on normal or pathological conditions in the brain.

Our study also reveals compelling evidence that KIBRA levels in CSF are associated with tau biomarkers and cognition in AD. These results point toward KIBRA as a biomarker for synapse dysfunction in tauopathy that could be useful for diagnosis and staging disease progression. The higher KIBRA levels detected in CSF may be linked to the downregulation of KIBRA in the synaptic compartment in tauopathies. For example, KIBRA may be expelled from neurons with dysfunctional synapses and accumulate in the CSF, but the mechanisms underlying the changes in KIBRA levels at synapses and in CSF are unclear. Our findings are consistent with prior studies showing higher SNAP-25, synaptotagmin-1, GAP-43, and neurogranin levels in CSF are associated with cognitive impairment in AD (33–35), which may be linked to synapse degeneration in the brain (72). To advance clinical application and potentially identify individuals that could benefit from a KIBRA therapeutic, future studies should focus on evaluating KIBRA as a biomarker of synapse dysfunction and cognitive decline together with engineering a CT-KIBRA–based therapeutic that can be delivered across the blood-brain barrier into the brain. CT-KIBRA–mediated synapse repair could be a valuable approach in combination with pathology-modifying strategies designed to slow progression of cognitive decline by reducing tau levels or clearing toxic forms of tau from the brain, which are being tested in ongoing clinical trials (73, 74). Our work supports a KIBRA-based synapse repair therapy that could promote the recovery of cognition in tauopathy with treatment by boosting the resilience of synapse function.

## Methods

### Mice

TauKQ^high^ mice (Gladstone Institutes) were previously generated (9), and maintained in the C57BL/6J genetic background. TauKQ^high^ mice were crossed with FVB/N mice (Jackson Laboratories) to generate tauKQ^high^ mice in an FVB/N and C57BL/6J mixed background for experiments. Behavior experiments were performed in daylight hours. Mice were housed in a pathogen-free barrier facility with a 12 hour light-dark cycle and provided with ad libitum access to water and food. Female and male mice were used in all experiments.

### Chemical LTP and IHC

Chemical LTP experiments were performed at 14–15 days in vitro (DIV) in extracellular solution (ECS) containing (in mM): 125 NaCl, 5 KCl, 25 HEPES, 1 NaH_2_PO_4_, and 11 Glucose, and 2.5 CaCl_2_ (all from Sigma-Aldrich); 0.0005 tetrodotoxin (Abcam); 0.1 picrotoxin (Caymen Chemicals); and 0.001 strychnine (Sigma-Aldrich; pH 7.4) warmed to 37°C. Rat hippocampal neurons were washed in ECS then incubated with 300 μM Glycine (Sigma-Aldrich) in ECS for 5 minutes at room temperature to induce chemical LTP while unstimulated neurons were washed with ECS. All neurons were washed with ECS and incubated for 10 minutes at 37°C. Coverslips (Carolina Biological)were treated with anti-GluA1 antibody (MilliporeSigma) in ECS for 15 minutes at 37°C then washed with ECS and fixed in 4% paraformaldehyde (PFA; Electron Microscopy Sciences). Coverslips were washed 3 times in PBS (Corning) then incubated in blocking solution containing PBS, 2% normal goat serum (Jackson ImmunoResearch), and 0.1% Triton X-100 (Sigma-Aldrich) for 1 hour. An anti–rabbit Alexa Fluor 647 (A21245, Invitrogen) secondary antibody was mixed in blocking solution and applied for 1 hour. For experiments using GFP-tagged proteins, neurons were labeled with anti-GFP conjugated to Alexa Fluor 488. For immunolabeling of flag-tagged proteins, cells were fixed in 4% PFA, washed 3 times with PBS, then incubated in PBS with 0.1% Triton X-100 for 5 minutes. Cells were washed twice in PBS, and blocked for 10 minutes in PBS with 1% BSA. Antibodies were added in PBS with 1% BSA for 1 hour, followed by 3 PBS washes. All coverslips were mounted on glass slides in Prolong Gold (Invitrogen). Images of transfected neurons were acquired on a Zeiss LSM 700 confocal microscope. Laser power and gain settings were kept constant across neurons within an experiment. Settings were established to keep the brightest pixel intensities just below saturation, except when the morphology of dendritic spines had to be clearly defined (e.g., saturated pixels of mApple fluorescence in dendrites to detect the signal in spines). For the analyses of immunolabeling within spines, the integrated intensity of GluA1, PKMζ, HA, or flag immunolabeling was measured within individual dendritic spines, which were outlined based on the spine morphology defined by either the mApple or GFP expressed in neurons. The intensities were analyzed from at least 50 spines per neuron using ImageJ software. Analysis was performed blind to the experimental condition.

### Western blot

A dounce homogenizer was used to homogenize human brain tissue samples in RIPA buffer (50 mM Tris [Invitrogen], pH 7.5, 150 mM NaCl [Sigma-Aldrich], 0.5% Nonidet P-40 [Thermo Fisher Scientific], 1 mM EDTA [MilliporeSigma]) containing 0.5% sodium deoxycholate (Sigma-Aldrich), 0.1% SDS (Invitrogen), 1 mM phenylmethyl sulfonyl fluoride (Sigma-Aldrich), protease inhibitor cocktail (Sigma-Aldrich), phosphatase inhibitor cocktail (Sigma-Aldrich), 5 mM nicotinamide (Sigma-Aldrich), and 1 μM trichostatin A (Sigma-Aldrich). Homogenized tissue was sonicated 20 times with 1 second pulses and centrifuged using a SW55 Ti rotor (Beckman Coulter) at 221,171*g* for 15 minutes at 4°C. Mouse tissue was homogenized in RIPA buffer containing 1 mM phenylmethyl sulfonyl fluoride, protease inhibitor cocktail (Sigma-Aldrich), phosphatase inhibitor cocktails 2 (Sigma-Aldrich) and 3 (Sigma-Aldrich). Tissue was homogenized with a hand-held homogenizer for 2 minutes and sonicated 10 times with 1 second pulses. Lysates were then incubated on ice for 30 minutes and centrifuged at 18,000*g* at 4°C for 20 minutes. The supernatants were collected from human or mouse brain homogenates after centrifugation and the protein concentration was measured by Bradford Assay (Bio-Rad). Equal amounts of proteins were run on a 4%–12% gradient SDS-PAGE gel (Invitrogen) and the protein was transferred to a nitrocellulose membrane (GE Healthcare). The membranes were blocked with 5% nonfat dry milk in TBST at room temperature for 1 hour and incubated with primary antibodies in TBST with 2% nonfat dry milk overnight at 4°C. Membranes were washed with TBST and incubated with either anti–rabbit horseradish peroxidase (111-035-144, Jackson ImmunoResearch) or anti–mouse horseradish peroxidase (115-035-166, Jackson ImmunoResearch) secondary antibodies in TBST with 2% nonfat dry milk at room temperature for 1 hour. After washing, chemiluminescence (Pierce) was used to detect immunolabeled proteins and ImageJ software (NIH) was used for quantification. Experiments that required quantification of immunoblots from different gels were done in parallel and 2–3 of the samples were run in duplicate on all gels. Quantification of immunolabeling of the duplicate samples on each immunoblot was then used to normalize the immunoblot analyses across all of the immunoblots. To calculate the ratio of acetylated tau or phosphorylated tau to total soluble tau in human brain homogenates, the immunoblotting of MAb359 or AT270 was performed first, then the immunoblots were stripped and reprobed with HT7. Immunoblots were stripped for 1 hour at 50°C in buffer containing 62.5 mM Tris pH 6.8, 2% SDS, and 0.71% β-mercaptoethanol. For each sample, MAb359 or AT270 immunoblot quantification was normalized to the respective reprobed HT7 immunoblot from the same membrane.

### IHC

Mice were perfused transcardially with cold PBS and half brains were fixed in cold 4% PFA for 48 hours. After fixation, brains were washed once in PBS and stored at 4°C. Brains were dehydrated for at least 48 hours in 30% sucrose with PBS then sectioned on a Leica SM2010R microtome. Standard staining procedure used mouse brain sections (30 μm) first washed with PBS and then blocked with 10% normal goat serum in PBS-T (0.5% Triton X-100) for 1 hour at room temperature. Primary antibodies were applied in PBS-T with 3% normal goat serum overnight at 4°C. After PBS-T washes, anti–rabbit Alexa Fluor 647 (A21245, Invitrogen) or anti–mouse Alexa Fluor 488 (A11029, Invitrogen) were applied in PBS-T with 3% normal goat serum for 1 hour at room temperature. For AT8 (1:1,000) immunostaining, sections were washed with PBS, heated to 95°C for 5 minutes in 10 mM Sodium Citrate and incubated at room temperature for 20 minutes before the blocking step. Images with AT8 staining were acquired on a digital microscope (KEYENCE) using a ×20 objective lens. For NeuN (1:1,000) immunostaining, sections were first washed with PBS, heated to 60°C for 60 minutes in Tris-EDTA pH 9.0 then followed by the standard staining procedure. Images of a region in CA1 and dentate gyrus were analyzed for NeuN positive cells using the IdentifyPrimaryObjects module from CellProfiler analysis software (version 4.2.5). Synapse density measurements were calculated from images of SV2 (1:500) and PSD-95 (1:50) antibody staining in CA1 stratum radiatum analyzed with ImageJ plugin JACoP (Just Another Co-localization Plugin). Images of SV2, PSD-95, and NeuN immunostaining were acquired using either ×20 or ×63 objectives on a LSM700 (Zeiss) confocal microscope. ImageJ software (NIH) was used to quantify immunofluorescence from AT8, PSD95, and SV2 immunostaining.

### PLA

HEK293 cells (Invitrogen) were plated on glass coverslips coated with poly L-lysine. Cells were transfected 24 hours later with plasmid DNA with Lipofectamine 2000 (Invitrogen). HEK cells were fixed in 4% PFA 24 hours after transfection and washed 3 times with PBS. Following the manufacturers protocol, coverslips were blocked in Duolink blocking solution (Sigma-Aldrich) and labeled with primary antibodies and the secondary antibodies, anti-Rabbit PLUS (DUO92002, Sigma-Aldrich) and anti-Mouse MINUS (DUO92004, Sigma-Aldrich). PLA signals were detected using the red Duolink in situ detection reagent kit (Sigma-Aldrich) and imaged on the Zeiss LSM700. Images were analyzed using ImageJ software.

### Protein stability assay

Plated HEK293 cells were transfected with HA-PKMζ or HA-PKMζ and CT-KIBRA-flag using Lipofectamine 2000. A day after transfection, untreated cells (0 hour time point) were harvested, while the rest of the cells were treated with 5 mg/mL of CHX. Treated cells were harvested after 24 and 48 hours of incubation with cycloheximide. Cells were resuspended in RIPA buffer containing 1 mM phenylmethyl sulfonyl fluoride, protease inhibitor cocktail (Sigma-Aldrich) and phosphatase inhibitor cocktails 2 (Sigma-Aldrich) and 3 (Sigma-Aldrich). Resuspended cells were sonicated and the supernatant was prepared the same as mouse tissue, then Western blot was performed to detect PKMζ and KIBRA levels.

### Human subjects

#### Brain tissue samples.

Tissue samples were acquired from the Neurodegenerative Disease Brain Bank at the University of California San Francisco. Neuropathological diagnoses were made following consensus diagnostic criteria (75, 76), and previously described histological and IHC methods were used (77, 78). Cases were selected based on clinical and neuropathological diagnoses. Frozen human brain tissue samples were dissected from the middle temporal gyrus of control (*n* = 6), AD (*n* = 7), and Pick’s disease (*n* = 7) cases.

#### CSF Samples.

For in vivo CSF KIBRA quantification, 25 older adults who were either clinically normal (*n* = 15) or diagnosed with AD (*n* = 10) were included. All participants were community-dwelling older adults who completed comprehensive neurologic, neuropsychological evaluations and a study partner interview at the University of California San Francisco Memory and Aging Center. Participants were reviewed and determined to be either within normative standards or meet research consensus criteria for AD (79) at a multidisciplinary consensus conference by board-certified neurologists and neuropsychologists. All clinically normal adults evidenced no functional decline (CDR = 0), while participants with AD ranged from mild to moderate stages of functional impairment (CDR range of 0.5–2).

CSF was collected via lumbar puncture in the morning following a 12-hour fast in sterile polypropylene tubes. Within 30 minutes of collection, CSF samples were centrifuged at 2000*g* at room temperature (20–25°C) for 5 minutes before being aliquoted into 500μL cryovials and stored at –80°C until analysis, following standard procedure (80).

### Statistics

Differences between 2 means were analyzed by 2-tailed Student’s *t* test, and differences between multiple means were analyzed by 1-way or 2-way ANOVA with Bonferroni multiple comparison post hoc analyses. Repeated measures 2-way ANOVA with Bonferroni multiple comparison was used to analyze input output results from field recordings of dentate gyrus and to analyze spatial learning in the MWM test. ClueGO analyses of mass spectrometry results showed networks with *P* values under 0.05 with right-sided hypergeometric testing and Bonferroni adjustment. Either Spearman’s’s or Pearson’s correlational analyses were used to evaluate the relationship between 2 variables. *P* values under 0.05 are considered significant.

### Study approval

All animal care and use were approved by the Buck Institute Research on Aging Institutional Animal Care and Use Committee (Novato, California, USA). For studies on human brain tissue, prior to autopsy, patients or their surrogates provided informed consent for brain donation, in keeping with the guidelines put forth in the Declaration of Helsinki. All study procedures for human CSF samples were also conducted in accordance with the latest Declaration of Helsinki and approved by the local UCSF Institutional Review Board. Participants provided written informed consent to participate in study procedures.

### Data availability

Data values are provided in the Supporting Data Values file. Complete mass spectrometry data sets have been uploaded to the Center for Computational Mass Spectrometry, to the MassIVE repository at UCSD, and can be downloaded using the following link: ftp://MSV000090510@massive.ucsd.edu (MassIVE ID no.: MSV000090510; ProteomeXchange ID: PXD037299).

## Author contributions

TET, GK, KAPN, KBC, and LG conceived the project and designed experiments. GK, KAPN, TET, LY, JHC, IW, and BS performed experiments. GK, KAPN, TET, KBC, LY, BS, and RS analyzed data. TCS, ALN, SS, LTG, WWS, and JHK provided experimental reagents. JHC, IW, HC, SS, YL, and DL provided technical support. TET, GK, KAPN, and KBC wrote the manuscript. The order of authorship was determined by overall contributions and approved by the authors for the final manuscript.

## Supplementary Material

Supplemental data

Unedited blot and gel images

Supplemental table 3

Supporting data values

## Version 1 02/01/2024

Electronic publication
