# Supplementary material for: KIBRA repairs synaptic plasticity and promotes resilience to tauopathy-related memory loss
Source: J Clin Invest. 2024 Feb 1;134(3):e169064. doi: 10.1172/JCI169064 (PMC10836803; doi:10.1172/JCI169064)
Supplement: Supplemental data [file jci-134-169064-s028.pdf]

Supplemental Data and Methods for

KIBRA repairs synaptic plasticity and promotes resilience to tauopathy-related memory loss

Grant Kauwe[1#], Kristeen A. Pareja-Navarro[1#], Lei Yao[1], Jackson H. Chen[1], Ivy Wong[1], Rowan Saloner[2], Helen Cifuentes[1], Alissa L. Nana[2], Samah Shah[1], Yaqiao Li[3], David Le[3], Salvatore Spina[2], Lea T. Grinberg[2,4], William W. Seeley[2,4], Joel H. Kramer[2], Todd C. Sacktor[5], Birgit Schilling[1], Li Gan[6], Kaitlin B. Casaletto[2], Tara E. Tracy[1]

[1] Buck Institute for Research on Aging, Novato, CA USA

[2] Memory and Aging Center, Department of Neurology, University of California San Francisco, San Francisco, CA USA

[3] Gladstone Institutes, San Francisco, CA USA

[4] Weill Institute for Neurosciences, Department of Pathology, University of California San Francisco, San Francisco, CA USA

[5] The Robert F. Furchgott Center of Neural and Behavioral Science, Departments of Physiology and Pharmacology, Anesthesiology, and Neurology, State University of New York Health Sciences University, Brooklyn, NY USA

[6] Helen and Robert Appel Alzheimer Disease Research Institute, Brain and Mind Research Institute, Weill Cornell Medicine, New York, NY USA

#Equal contributions

Correspondence:
Tara E. Tracy, PhD
Buck Institute for Research on Aging
8001 Redwood Blvd, Novato CA 94947
Phone: 415-209-2062
Email: ttracy@buckinstitute.org
Conflict of interest: The authors declare no conflict of interest exists.

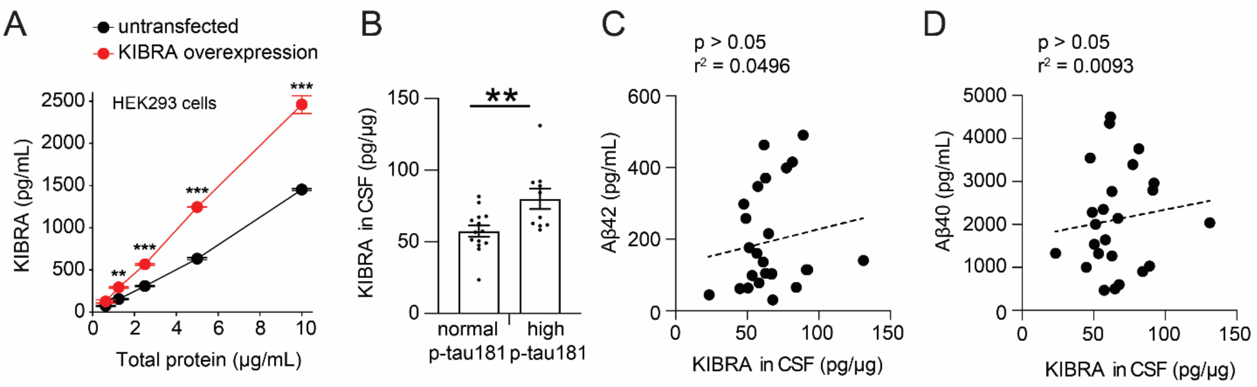

Supplemental Figure 1. Human KIBRA detection by ELISA and CSF KIBRA relative to phosphorylated tau and Aβ related to Figure 1.

(A) ELISA-based detection of KIBRA levels from lysates of HEK293 cells transfected with a human KIBRA construct (red) and untransfected control HEK293 cells (black). HEK293 cells with KIBRA overexpression had significantly higher KIBRA levels detected by ELISA at the different concentrations of total protein tested (n = 2 cultures/group; $^{**}p < 0.01$, $^{***}p < 0.001$, two-way ANOVA, Bonferonni *post*-hoc analyses). Values are given as means ± SD.

(B) Quantification of the concentration of KIBRA in total protein detected in cerebrospinal fluid (CSF) of human subjects that had either normal (< 61 pg/mL) or high (> 61 pg/mL) levels of phosphorylated tau (p-tau181) in CSF associated with Alzheimer's disease ($^{**}p < 0.01$, Student's *t*-test). Values are given as means ± SEM.

(C and D) Spearman correlation analyses show the relationship between levels of KIBRA with concentrations of **(C)** Aβ42 and **(D)** Aβ40 in CSF (n = 24 subjects).

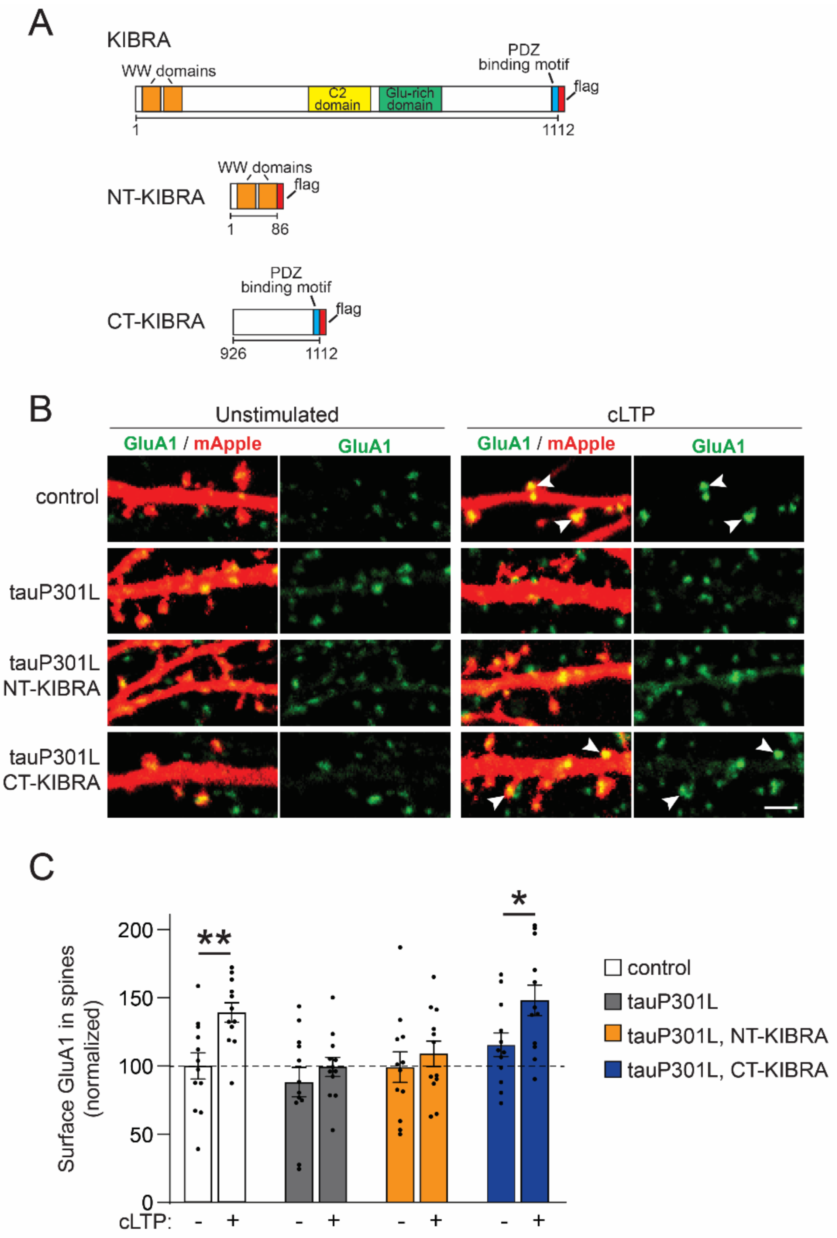

Supplemental Figure 2. CT-KIBRA rescues AMPAR trafficking during LTP in neurons expressing human tau with the P301L mutation that causes FTD related to Figure 2.

(A) Design of flag-tagged KIBRA constructs.

(B) Representative confocal images of surface GluA1 immunolabeling (green) in spines on cultured hippocampal neurons expressing mApple (red) with or without tauP301L and one of the KIBRA variants. Scale bar, 2 μm.

(C) Quantification of surface GluA1-containing AMPARs in spines revealed that tauP301L expression in neurons inhibited chemical LTP (cLTP)-induced postsynaptic AMPAR insertion, which was restored by the co-expression of CT-KIBRA (n = 12 neurons/group; $*p < 0.05$, $**p < 0.01$, unpaired Student's *t*-test). Values are given as means ± SEM.

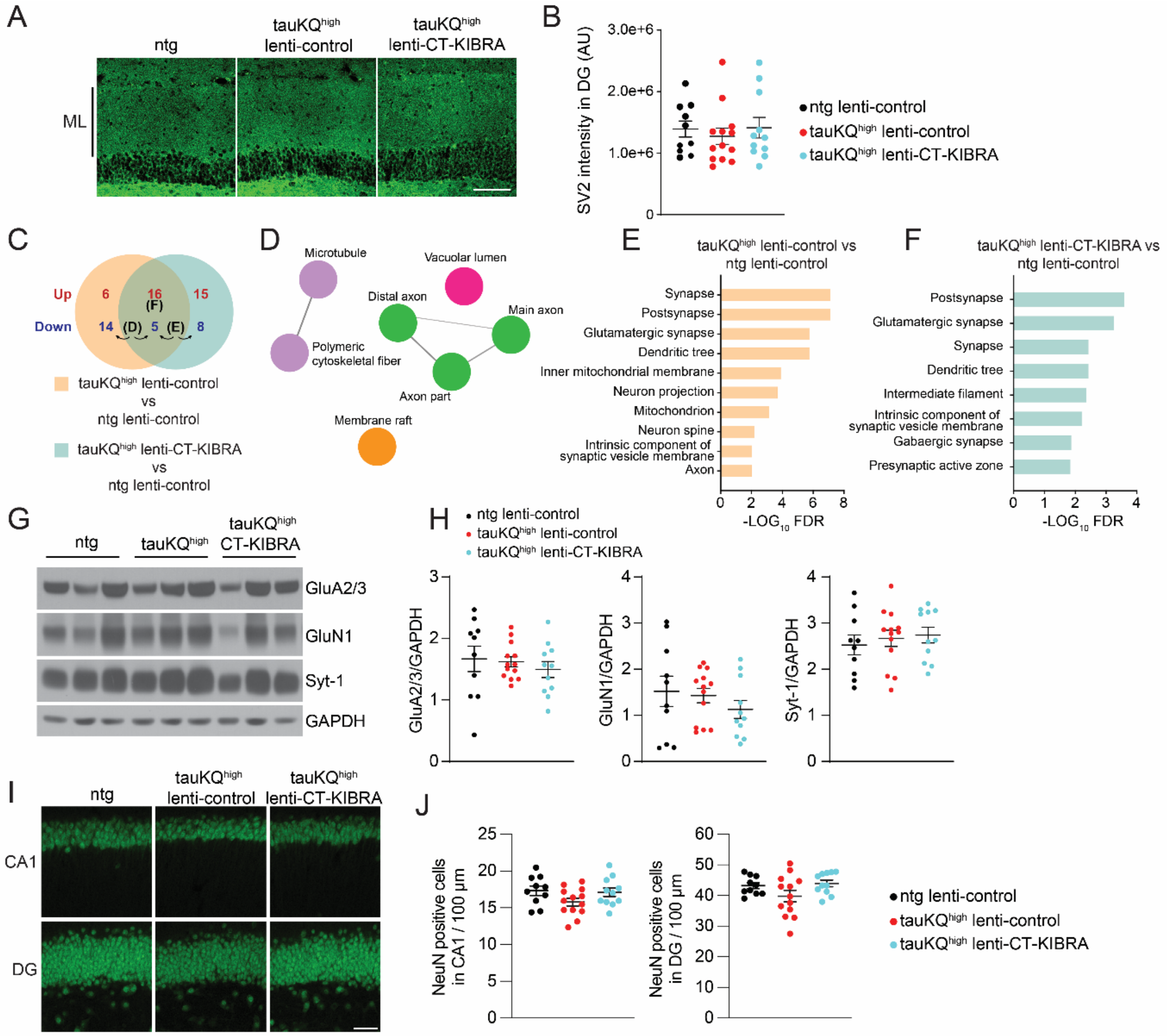

Supplemental Figure 3. Synapse and proteomics analyses of hippocampus from tauKQ^{high} mice with or without CT-KIBRA expression related to Figure 4.

(A) Representative confocal images of SV2 immunolabeling as a marker of synapses in the molecular layer (ML) of the dentate gyrus (DG). Scale bar, 100 µm.

(B) Quantification of the mean integrated intensity of SV2 immunofluorescence in the ML of the DG (n = 10-13 mice/group; $p > 0.05$, one-way ANOVA, Bonferonni post-hoc analyses). Values are given as means ± SEM.

(C) Venn diagram depicting the number of proteins identified that were either upregulated or downregulated in tauKQ^{high} lenti-control or tauKQ^{high} lenti-CT-KIBRA compared to non-transgenic (ntg) control mice (n = 4 mice/group). See also Supplemental Table 3.

(D) ClueGO cellular component enrichment of proteins that were upregulated in hippocampus of tauKQ^{high} mice with or without CT-KIBRA expression compared to ntg lenti-control mice. Node colors denote grouped networks ($p < 0.0005$).

(E and F) Gene Set Enrichment Analysis (GSEA) on the proteins identified that were downregulated in **(E)** tauKQ^{high} lenti-control mice and in **(F)** tauKQ^{high} lenti-CT-KIBRA mice compared to controls. Downregulated synaptic proteins in tauKQ^{high} mice with or without CT-KIBRA included Synapsin-1 and SynGAP.

(G and H) Immunoblot analyses and quantification of glutamate receptor subunits and Synaptotagmin-1 (Syt-1) in whole hippocampus lysates (n = 10-13 mice/group; $p > 0.05$, one-way ANOVA).

(I) Representative confocal images of NeuN immunolabeling in CA1 and DG. Scale bar, 100 µm.

(J) Quantification of NeuN positive cells identified by CellProfiler™ in CA1 and DG per 100 µm (n = 10-13 mice/group; $p > 0.05$, one-way ANOVA). Values are given as means ± SEM.

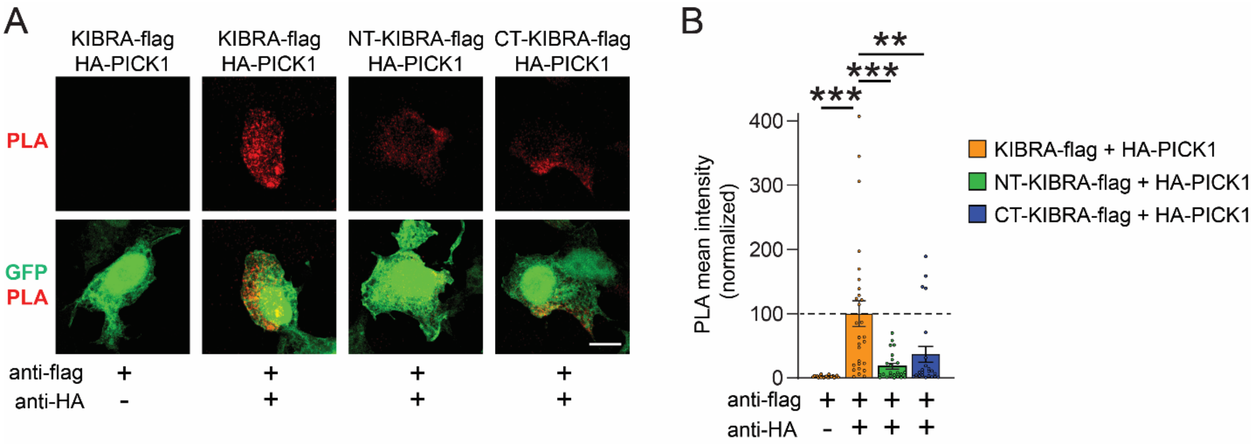

Supplemental Figure 4. PICK1 interacts more with full-length KIBRA than the truncated KIBRA variants related to Figure 5.

(A) HEK293 cells were transfected with GFP (green), HA-tagged PICK1 and either flag-tagged full-length KIBRA, NT-KIBRA or CT-KIBRA constructs. The proximity ligation assay (PLA) was performed with anti-flag and anti-HA antibodies to detect the PICK1 and KIBRA interacting within close proximity (red). Scale bar, 10 µm.

(B) Graph of mean PLA intensity quantification in HEK293 cells normalized to PLA signal in cells with full-length KIBRA (n = 22-28 cells/group; **$p < 0.01$, ***$p < 0.001$, one-way ANOVA, Bonferonni post-hoc analyses). Values are given as means ± SEM.

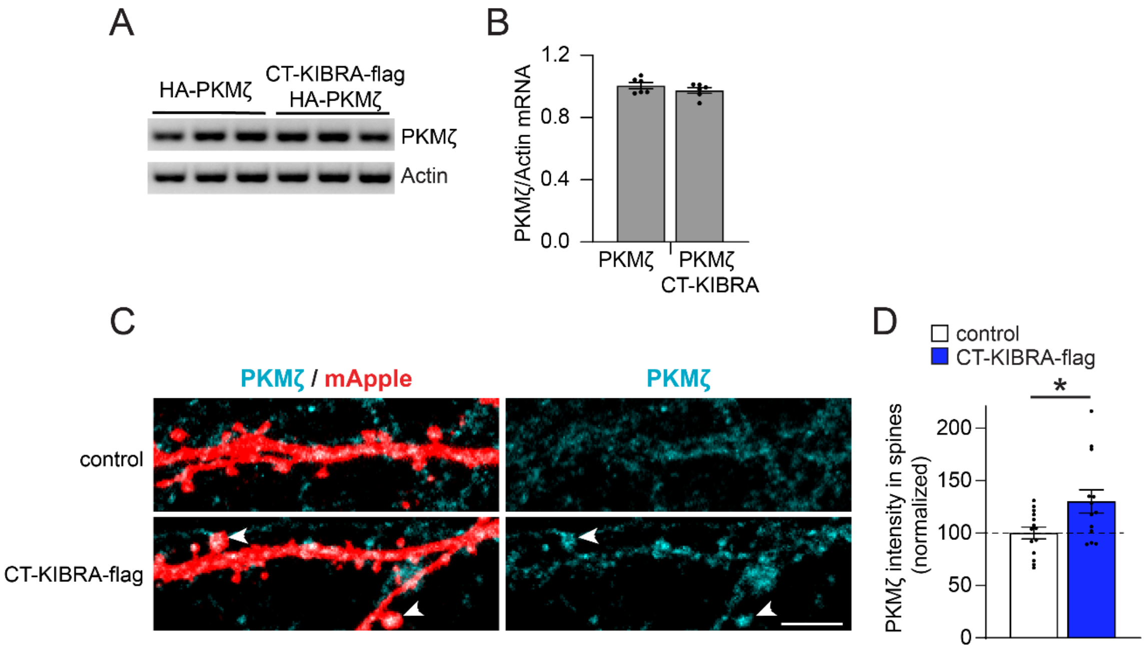

Supplemental Figure 5. Analyses of PKMζ levels in HEK293 cells and neurons with CT-KIBRA expression related to Figure 5.

(A) Representative agarose gel electrophoresis of RT-PCR products from HEK293 cells transfected with either HA-PKMζ or HA-PKMζ and CT-KIBRA-flag.

(B) Quantification of the intensity of amplified DNA bands reverse transcribed from *PKMζ* mRNA obtained from HEK293 cells shown in **(A)** (n = 6; no significant differences between groups, unpaired Student's *t*-test).

(C) PKMζ immunolabeling (cyan) in cultured hippocampal neurons with mApple expression (red) with or without CT-KIBRA. Postsynaptic spines contained PKMζ (arrowheads). Scale bar, 5 μm.

(D) Quantification of PKMζ levels in postsynaptic spines of hippocampal neurons with or without CT-KIBRA expression (n = 13-14 neurons/group; * $p < 0.05$, unpaired Student's *t*-test). Values are given as means ± SEM.

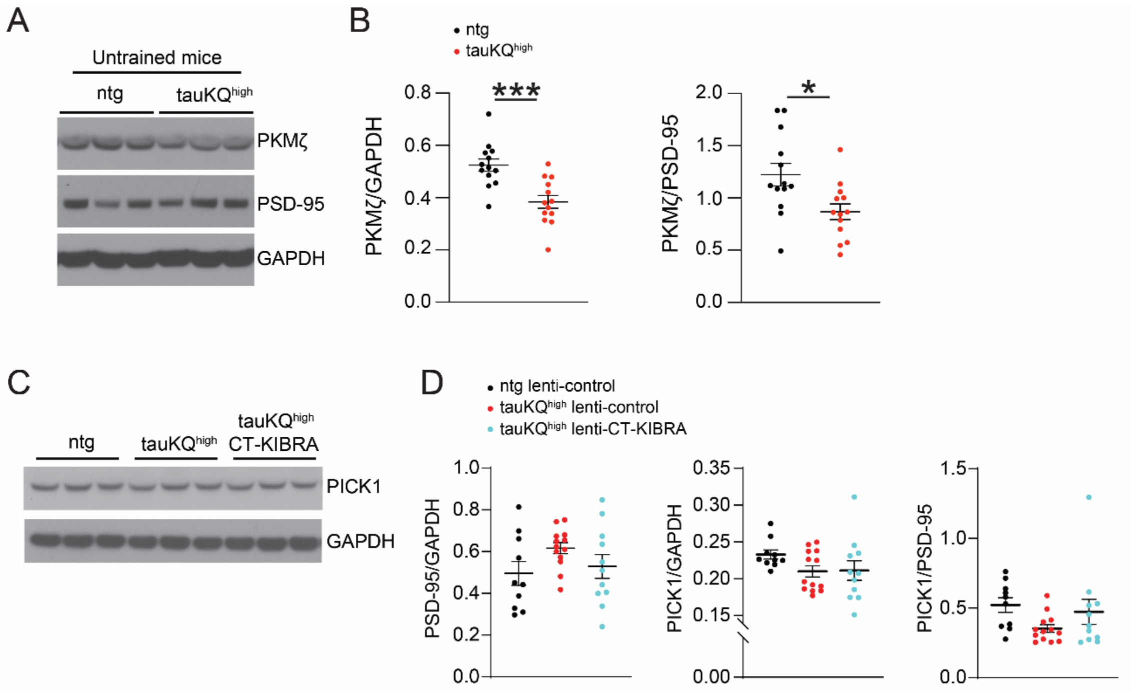

Supplemental Figure 6. Levels of PKMζ and other synaptic proteins in hippocampus of tauKQ^{high} mice related to Figure 6.

(A) Immunoblots of PKMζ, PSD-95 and GAPDH from hippocampal lysates of three non-transgenic (ntg) and three tauKQ^{high} mice that did not receive training in behavioral tests of hippocampus-dependent learning and memory.

(B) Graphs of PKMζ levels relative to GAPDH and PSD-95 in mice that did not have training in behavioral tests (n = 13 mice/group; *p < 0.05, ***p < 0.001, unpaired Student's *t*-test).

(C) Immunoblots of PICK1 and GAPDH from hippocampal lysates of three mice from ntg lenti-control, tauKQ^{high} lenti-control, and tauKQ^{high} lenti-CT-KIBRA groups that underwent training in learning and memory tests. A representative PSD-95 immunoblot is shown in Figure 6C.

(D) Quantification of PSD-95 levels and PICK1 levels in hippocampal lysates from immunoblots (n = 10-13 mice/group; no significant differences between groups, one-way ANOVA). PICK1 levels were also normalized to PSD-95. Values are given as means ± SEM.

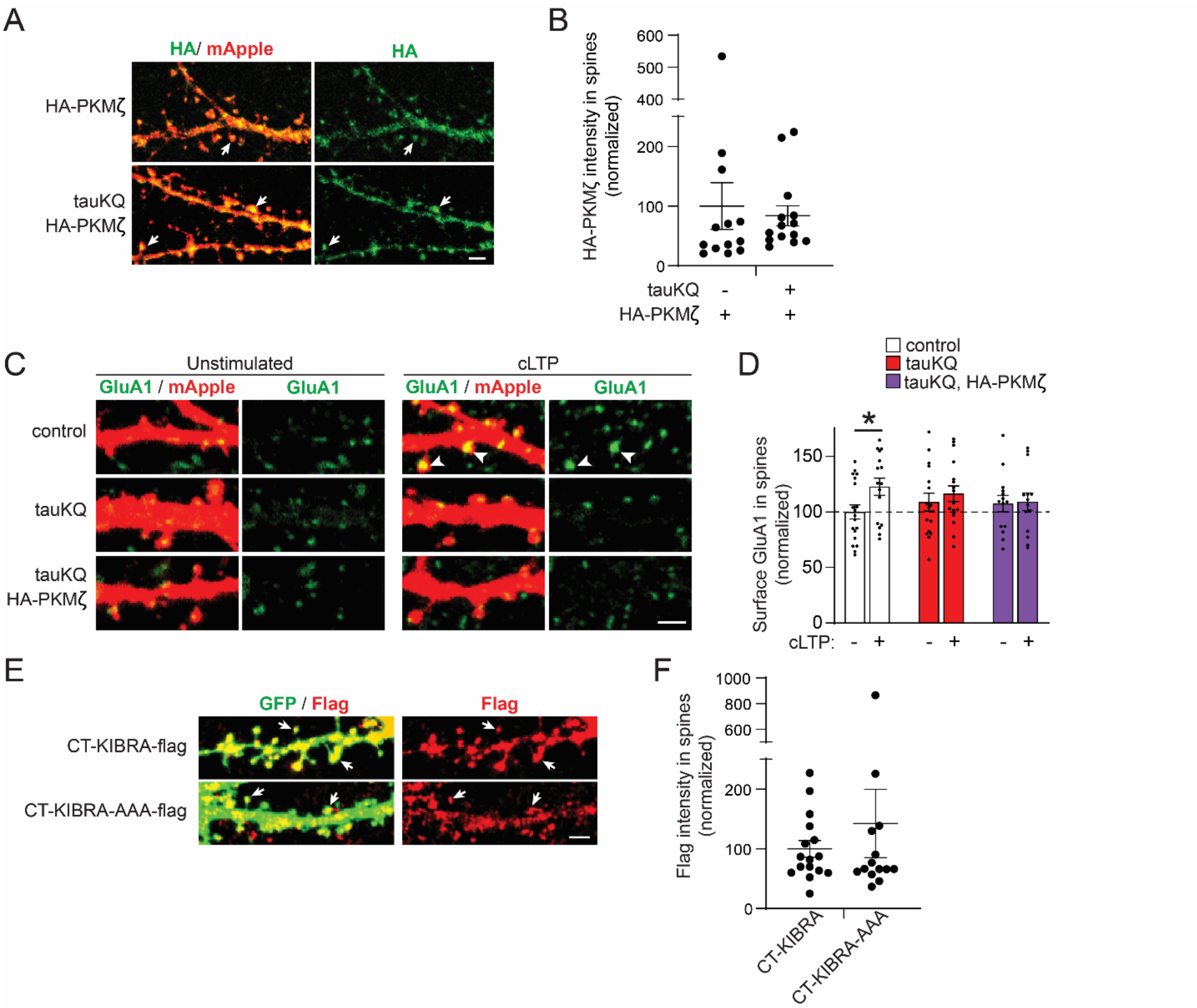

Supplemental Figure 7. Expression of HA-PKMζ and CT-KIBRA constructs in cultured hippocampal neurons related to Figure 7.

(A) Representative confocal images of anti-HA immunolabeling (green) of HA-tagged PKMζ overexpressed in neurons with mApple (red). HA-PKMζ was detected in spines (arrows). Scale bar, 2 μm.

(B) Quantification of the mean intensity of HA-tagged PKMζ immunolabeling in spines normalized to neurons without tauKQ co-expression (n = 13-14 neurons/group).

(C) Representative confocal images of dendrites and spines on neurons expressing mApple (red) alone, mApple with tauKQ, or mApple with tauKQ and HA-PKMζ that were unstimulated or subjected to cLTP followed by surface GluA1 immunolabeling (green). Scale bar, 2 μm.

(D) Quantification of the surface GluA1 immunofluorescence in spines normalized to unstimulated control neurons (n = 14-19 neurons/group; *$p < 0.05$, unpaired Student's t-test).

(E) Representative confocal images of GFP-expressing neurons (green) with co-expression of flag-tagged CT-KIBRA or CT-KIBRA-AAA immunostained with an anti-flag antibody (red). CT-KIBRA with or without the three alanine mutations was detected in spines (arrows). Scale bar, 2 μm.

(F) Quantification of the mean intensity of flag immunofluorescence detected in spines of neurons with CT-KIBRA-AAA compared to CT-KIBRA (n = 14-16 cells/group). Values are given as means ± SEM.

Supplemental Table 1. Patient information for human brain tissues used in this study.

Case number	Sex	Age at death	Clinical	Primary Neuropath Dx	PMI (hrs)[a]	ADNC[b]	CDR	CDRsum
P2323.10	M	70	bvFTD	Pick's	10.2	-	x	x
P2494	M	72	bvFTD	Pick's	24.3	Not ADNC	x	x
P2533	F	76	bvFTD	Pick's	25.5	Not ADNC	3	18
P2682	F	74	nfvPPA	Pick's	9.8	Not ADNC	1	5.5
P2839	M	86	AD, Vascular	Pick's	14.8	Low	3	18
P3043	F	76	nfvPPA	Pick's	5.9	Low	x	x
P3052	M	78	svPPA (right)	Pick's	7.5	Not ADNC	3	17
P2345.11	F	86	control	none	7.8	Low	x	x
P2577	M	94	control	none	20	Low	x	x
P2594	F	86	control	none	6.4	Not ADNC	3	13
P2850	F	92	control	none	8	Not ADNC	1	5.5
P2921	F	82	control	none	9.6	Low	x	x
P3045	F	84	control	none	7.7	Low	0	0
P2412.12	M	88	AD	AD	10.8	High	x	x
P2484	M	88	AD	AD	9.8	High	x	x
P2508	M	82	AD	AD	6.7	High	3	16
P2554	M	85	AD	AD	11.2	High	1	6
P2592	M	86	AD	AD	8.6	High	2	12
P2612	M	82	AD	AD	9	High	3	18
P2823	F	89	AD	AD	15.8	High	3	18

[a]PMI = Post-mortem interval

[b]ADNC = Alzheimer's disease neuropathological change

Supplemental Table 2. Patient information for the cerebrospinal fluid (CSF) used in this study.

Case number	Age	Sex	Diagnosis	MMSE[a]
2720	80	F	normal	28
5064	58	M	normal	x
7261	70	M	normal	29
7841	69	F	normal	30
8843	70	M	normal	29
12955	66	M	AD	18
13690	62	M	AD	24
14744	65	M	AD	26
15035	69	F	AD	30
15313	74	F	normal	29
15468	57	M	AD	23
15508	60	F	normal	x
15590	68	F	AD	21
15791	56	F	AD	25
16304	60	F	AD	8
17412	63	M	AD	12
17615	53	M	normal	x
17735	71	F	normal	30
18176	55	M	normal	x
21514	68	F	normal	30
25992	54	F	normal	29
26161	80	M	AD	25
26234	72	M	normal	30
26323	55	M	normal	30
29442	69	M	normal	29

[a]MMSE = Mini-Mental State Examination

SUPPLEMENTAL METHODS

ELISAs

Commercially available enzyme-linked immunosorbent assay (ELISA) kits were used to measure levels of KIBRA (Wuhan Fine Biotech Co., Ltd, China), p-tau181, t-tau (INNOTEST, USA), Aβ40 and Aβ42 (ThermoFisher, USA) in CSF samples. ELISAs on CSF were performed in duplicates and averaged. Samples with % CV > 20% were excluded from analyses.

Cultured Neurons

Primary hippocampal neurons were harvested from embryonic day 22 pups extracted from timed pregnant female rats (Charles River). Neurons were plated on coverslips coated with poly-L-lysine (Sigma) in Neurobasal medium with B27 supplement (Gibco) and Glutamax (Invitrogen). Half the media was replaced at 1 day in vitro (DIV). At 7 DIV, half the media was replaced and 4 μM cytosine β-D-arabinofuranoside (Sigma) was added. Neurons were transfected with plasmids using Lipofectamine 2000 (Thermo Fisher Scientific) at 10-11 DIV.

Plasmids

Flag-tagged full-length human KIBRA, NT-KIBRA and CT-KIBRA were cloned into the pCDNA 3.1 vector. The human KIBRA sequence originated from the pBabepuro-KIBRA vector (81) (Addgene #40887). For lentiviral-based expression, the flag-tagged CT-KIBRA sequence was inserted into FUGW2 (Gladstone Institutes) and packaged into virus using Δ8.9 and VSV-G plasmids. The KIBRA and CT-KIBRA sequences used were from a splice variant of KIBRA that lacks a Q residue in the KIBRA C-terminal domain (82). HA-tagged PKMζ and HA-tagged PICK1 were cloned into pcDNA 3.1 from human cDNA plasmids verified by sequencing (SinoBiological). GFP-tagged human tauKQ and tauP301L were expressed from the pEGFP-C1 plasmid (Clontech). Expression of mApple or GFP in cultured neurons was done using pGW1-

mApple and pEGFP-C1 plasmids, respectively. CT-KIBRA-AAA mutant was generated by site-directed mutagenesis of CT-KIBRA in pcDNA3.1 (Genscript).

Antisense Oligonucleotide

Antisense oligonucleotides against *PKMζ* (C*T*C*TTGGGAAGGCAT*G*A*C) and scrambled oligonucleotides (A*A*C*AATGGGTCGTCT*C*G*G) were generated (IDT) with phosphorothioate bonds (*). For chemical LTP experiments, rat neurons were preincubated with oligonucleotides (20 μM final) for 1 h before chemical LTP, and the oligonucleotides remained in the media for the duration of the chemical LTP experiment.

Stereotaxic Injection and Virus

Flag-tagged CT-KIBRA (residues 926 to 1112) was subcloned into a lentiviral FUGW2 plasmid. The FUGW2 plasmid containing CT-KIBRA was co-transfected with Δ8.9 and VSV-G packaging plasmids in HEK293 cells using calcium phosphate transfection. The lentivirus was propagated in HEK293 cells at 37°C and was collected twice over a 48-hour period. Collected media containing the lentivirus was purified using sucrose gradient ultracentrifugation and resuspended in sterile PBS. Lentivirus titer was estimated using a p24 Rapid Titer Kit (Takara Bio USA, Inc). Mice were anesthetized by isoflurane inhalation, and a stereotax was used to position the lentivirus injection directly into the mouse hippocampus using the following coordinates from bregma: anterior-posterior: -2.0, medial-lateral ±1.5, dorsal-ventral -1.8. The lentivirus was injected into the hippocampus at a rate of 0.5 μL/min. Mice received analgesic treatment with buprenorphine with one dose at the start of the surgery and two additional doses within 24 h after surgery.

Acute Slice Preparation

Mouse brains were dissected into cold sucrose dissection solution containing (in mM): 210 sucrose, 2.5 KCl, 1.25 NaH2PO4, 25 NaHCO3, 7 glucose, 2 MgSO4, and 0.5 CaCl2 (perfused with 95% O2, 5% CO2 with ph ~7.4). Horizontal slices were cut at 400 μm thickness (VT1000S Vibratome, Leica) and incubated for 30 min in artificial ACSF at 35°C containing (in mM): 119 NaCl, 2.5 KCl, 26.2 NaHCO3, 1 NaH2PO4, 11 Glucose, 1.3 MgSO4 (gassed with 95% O2, 5% CO2 ph ~7.4). After recovery, slices were kept at room temperature with continuous oxygen bubbling.

Electrophysiology

Extracellular field recordings were performed from acute horizontal brain slices. Recording electrodes were placed in the molecular layer of the dentate gyrus. Slices were placed in recording chambers perfused with oxygenated ACSF solution heated to 30°C. Recording electrodes (~3 megaohms resistance) were filled with ACSF and lowered 50 μm into the dorsal blade of the molecular layer of the dentate gyrus. A bipolar tungsten electrode (FHC) was positioned ~150 μm away from the recording electrode to stimulate the perforant pathway inputs to the dentate gyrus. Stimulus pulses were elicited at an intensity range from 0.25 μA – 25 μA every 30 s with a 0.5 ms stimulus duration using a Model 2100 Isolated Pulse Stimulate (A-M Systems) to acquire the maximal fEPSP slope. The stimulus intensity was adjusted to 30% of the maximal fEPSP slope to record the baseline for LTP recordings. LTP recordings were performed in the presence of 100 μM picrotoxin (Sigma). Baseline fEPSPs were recorded for 20 min. Following the baseline, the stimulus intensity was increased to 60% of the maximal fEPSP slope for the theta burst stimulation (TBS) only. TBS consisted of 10 theta bursts applied every 15 s and each theta burst contained 10 bursts (4 pulses, 100 Hz) every 200 ms. After TBS the stimulus intensity was returned to the same level as during the baseline LTP recordings and fEPSPs were recorded for an additional 60 min. The fEPSP slope was normalized to baseline LTP responses. Recordings were acquired with

WinLTP software (version 1.11b, University of Bristol) using a Multiclamp 700B amplifier (Molecular Devices). Recordings and analyses were performed blind to genotype.

Behavioral Tests

For the object-context discrimination test, mice were habituated in the testing room while in their home cage for 30 min before the start of testing. During the sample phase, mice explored two similar, but distinct contexts in two different 10 min sessions that were 30 min apart. Context 1 contained two identical objects (X_1 and X_2) in a white box that was cleaned with 70% ethanol. Context 2 contained a different set of objects (Y_1 and Y_2) in a white box with black and white checkered wallpaper that was cleaned with 1% acetic acid. The mice were returned to their home cages for 4 h after the sample phase. For the test phase, one of the X objects in Context 1 was replaced with an incongruent Y object, and one of the Y objects in Context 2 was replaced with an incongruent X object. Half of the mice, balanced by genotype and sex, were tested in Context 1 and the other half were tested in Context 2. The amount of time mice spent exploring the congruent and incongruent objects during a 10-min session was calculated. Videos of the sessions were manually scored by an experimenter who was blind to mouse genotype and treatment.

Y-maze arena was constructed with plexiglass having three arms of equal length (20 cm) at equal angles. Mice were placed in the center and allowed to freely explore the arena for 5 min and recorded by video (Noldus). Each mouse was manually scored for the sequence and number of arm entries in the 5-min period and the experimenter was blind to the genotype and treatment of each mouse. An arm entry was defined as having all four paws within one arm. The percentage of spontaneous alternations for each mouse was calculated as the number of alternations divided by the total possible number of alternations in the 5-min session. An alternation is defined as the mouse having entered all three arms in succession without revisiting a previously entered arm.

The Morris water maze used a pool with a diameter of 120 cm and filled with water at a temperature 22 ± 1°C made opaque by the addition of white tempera paint. Visual cues were placed around the pool. The experimenter was blind to the genotype and treatment of each mouse. Mice were pretrained for one day with 4 trials to find a submerged hidden square platform (14 cm x 14 cm) that was 1.5 cm below the water surface located in the center of a rectangular channel. The day after pretraining, each mouse performed 4 days of hidden platform training with 2 trials per day where they had 60 s to find and sit independently on the platform for 10 s. Upon completion of hidden platform training, the platform was removed from the pool and mice were tested in a single probe trial for 60 s on subsequent days. Following probe trials, all mice were tested in cued platform training. Each mouse was recorded and tracked during hidden platform training, probe trials, and cued platform training using EthoVision XT software (Noldus).

Mass Spectrometry

Sample preparation. Mouse hippocampal tissue from 4 biological replicates for each group was homogenized in RIPA buffer (50 mM Tris-HCl pH 7.5, 0.5% Nonidet P-40, 150 mM NaCl, 1 mM EDTA, 1 mM phenylmethyl sulfonyl fluoride, protease inhibitor cocktail, and phosphatase inhibitors).

Proteolytic digestion. Aliquots of 150 µg protein for each tissue sample were subjected to lysis buffers containing 5% SDS and 50 mM triethylammonium bicarbonate (TEAB), at pH ~7.55. The samples were reduced in 20 mM dithiothreitol (DTT) in 50 mM TEAB for 10 minutes at 50º C, subsequently cooled at room temperature for 10 minutes, and then alkylated with 40 mM iodoacetamide (IAA) in 50 mM TEAB for 30 minutes at room temperature in the dark. Samples were acidified yielding a final concentration of 1.2% phosphoric acid, resulting in a visible protein colloid. Subsequently, 90% methanol in 100 mM TEAB was added at a volume of 7 times the

acidified lysate volume. Samples were vortexed until the protein colloid was thoroughly dissolved in the 90% methanol solution. The entire sample volume was spun through micro S-Trap columns (Protifi) collecting the flow-through in an Eppendorf tube (in 200 µL aliquots for 20 seconds at 4,000 x g), and importantly binding the samples to the S-Trap columns. Subsequently, the S-Trap columns were washed with 200 µL of 90% methanol in 100 mM TEAB (pH ~7.1) twice for 20 seconds each at 4,000 x g. S-Trap columns were placed into a clean elution tube and incubated for 1 hour at 47º C with 125 µL of trypsin digestion buffer (in 50 mM TEAB, pH ~8) at a 1:25 ratio (protease:protein, wt:wt). The same mixture of trypsin digestion buffer was added again for an overnight incubation at 37º C. Peptides were sequentially eluted from S-Trap micro spin columns with 50 mM TEAB, 0.5% formic acid (FA) in water, and 50% acetonitrile (ACN) in 0.5% FA.

Desalting. After centrifugal evaporation, samples were resuspended in 0.2% formic acid (FA) in water and desalted with Oasis 10-mg Sorbent Cartridges (Waters, Milford, MA). Samples were then subjected to an additional centrifugal evaporation and were finally re-suspended in 0.2% FA in water with a final concentration of 1 µg/µL. One microliter of indexed Retention Time Standard (iRT, Biognosys, Schlieren, Switzerland) was added to each sample.

Mass spectrometry system. Briefly, samples were analyzed by reverse-phase HPLC-ESI-MS/MS using an Eksigent Ultra Plus nano-LC 2D HPLC system (Dublin, CA) with a cHiPLC system (Eksigent) which was directly connected to a quadrupole time-of-flight (QqTOF) TripleTOF 6600 mass spectrometer (SCIEX, Concord, CAN). After injection, peptide mixtures were loaded onto a C18 pre-column chip (200 µm x 0.4 mm ChromXP C18-CL chip, 3 µm, 120 Å, SCIEX) and washed at 2 µl/min for 10 min with the loading solvent (H_2O/0.1% formic acid) for desalting. Subsequently, peptides were transferred to the 75 µm x 15 cm ChromXP C18-CL chip, 3 µm, 120 Å, (SCIEX), and eluted at a flow rate of 300 nL/min with a 3 h gradient using aqueous and acetonitrile solvent buffers.

Data-dependent acquisitions (for spectral library building): For peptide and protein identifications the mass spectrometer was operated in data-dependent acquisition (DDA) mode, where the 30 most abundant precursor ions from the survey MS1 scan (250 msec) were isolated at 1 m/z resolution for collision induced dissociation tandem mass spectrometry (CID-MS/MS, 100 msec per MS/MS, 'high sensitivity' product ion scan mode) using the Analyst 1.7 (build 96) software with a total cycle time of 3.3 sec as previously described (83).

Data-independent acquisitions: For quantification, all peptide samples were analyzed by data-independent acquisition (DIA, e.g., SWATH), using 64 variable-width isolation windows (84, 85). The variable window width is adjusted according to the complexity of the typical MS1 ion current observed within a certain m/z range using a DIA 'variable window method' algorithm (more narrow windows were chosen in 'busy' m/z ranges, wide windows in m/z ranges with few eluting precursor ions). DIA acquisitions produce complex MS/MS spectra, which are a composite of all the analytes within each selected Q1 m/z window. The DIA cycle time of 3.2 sec included a 250 msec precursor ion scan followed by 45 msec accumulation time for each of the 64 variable SWATH segments.

Mass-spectrometric data processing, quantification and bioinformatics. Mass spectrometric data-dependent acquisitions (DDA) were analyzed using the database search engine ProteinPilot (SCIEX 5.0 revision 4769) using the Paragon algorithm (5.0.0.0,4767). Using these database search engine results a MS/MS spectral library was generated in Spectronaut 14.2.200619.47784 (Biognosys). The DIA/SWATH data was processed for relative quantification comparing peptide peak areas from various different time points during the cell cycle. For the DIA/SWATH MS2 data sets quantification was based on XICs of 6-10 MS/MS fragment ions, typically y- and b-ions, matching to specific peptides present in the spectral libraries. Peptides were identified at Q< 0.01%, significantly changed proteins were accepted at a 5% FDR (q-value < 0.05). Differential expression analysis was performed using a paired t-test, and p-values were corrected for multiple testing, specifically applying group wise testing corrections using the Storey method (86). Gene

Ontology (GO) term enrichment analyses were performed with Gene Set Enrichment Analysis (GSEA) (87, 88) or ClueGO version 2.5.4 in Cytoscape version 3.7.1 (89, 90). Enrichment analyses were applied from GO Cellular Compartments.

RNA extraction and quantification

Each well was plated with 0.5 x 10^6 HEK293 cells in a 12-well culture plate. The cells were transfected with HA-PKMζ or HA-PKMζ and CT-KIBRA-flag using Lipofectamine 2000. A day after transfection, mRNA from transfected HEK293 cells were extracted using a Total RNA Isolation Kit (Thermofisher). iScript cDNA Synthesis Kit (BioRad) was used to obtain cDNA from mRNA. PCR was performed on cDNA using the *PKMζ* primer pairs: *PKMζ* 'CTT ACA TTT CCT CAT CCC GGA AG' and 'TTC ACC ACT TTC ATG GCG TAA A'. Actin primer pairs 'ACA CCC TTT CTT GAC AAA ACC T', and 'CGC ATC TCA TAT TTG GAA TGA CT' were used for normalization. PCR products were analyzed using agarose gel electrophoresis and ImageJ software (NIH) was used for quantification of the intensity of the bands.

Antibodies

Monoclonal antibodies used included: AT270 (MN1050, Thermo Fisher Scientific, RRID: AB_223651), AT180 (MN1040, Thermo Fisher Scientific, RRID: AB_223649), HT7 (MN1000, Thermo Fisher Scientific, RRID: AB_2314654), Tau5 (AHB0042, Thermo Fisher Scientific, RRID: AB_2536235), MAb359 (from Dr. Li Gan), anti-GAPDH (MAB374, Sigma-Aldrich, RRID: AB_2107445), anti-FLAG (F1804, Sigma-Aldrich, RRID: AB_262044), AT8 (MN1020, Thermo Fisher Scientific, RRID: AB223647), anti-SV2 (University of Iowa DSHB, RRID: AB_2315387), anti-PICK1 (75-040, Antibodies Inc, RRID: AB_2164544), anti-KIBRA (sc-133374, Santa Cruz Biotechnology, RRID:AB_2216359). Polyclonal antibodies used included: anti-PKMζ (from Dr. Todd C. Sacktor), anti-PSD95 (2507, Cell Signaling Technology, RRID: AB_561221), anti-HA (H6908, Sigma-Aldrich, AB_260070), GFP (A-21311, Thermo Fisher Scientific, RRID:

AB_221477), anti-GluA1 (ABN241, Millipore, RRID: AB_2721164). NeuN (ab177487, Abcam, RRID: AB_2532109), Rb Alexa-647 (A21245, Invitrogen, RRID: 2535813).

REFERENCES

81. Moleirinho S, et al. KIBRA exhibits MST-independent functional regulation of the Hippo signaling pathway in mammals. *Oncogene.* 2013;32(14):1821-30.
82. Ferguson L, et al. Isoform Specificity of PKMs during Long-Term Facilitation in Aplysia Is Mediated through Stabilization by KIBRA. *The Journal of neuroscience : the official journal of the Society for Neuroscience.* 2019;39(44):8632-44.
83. Christensen DG, et al. Identification of Novel Protein Lysine Acetyltransferases in Escherichia coli. *mBio.* 2018;9(5).
84. Collins BC, et al. Multi-laboratory assessment of reproducibility, qualitative and quantitative performance of SWATH-mass spectrometry. *Nature communications.* 2017;8(1):291.
85. Schilling B, et al. Generation of High-Quality SWATH(®) Acquisition Data for Label-free Quantitative Proteomics Studies Using TripleTOF(®) Mass Spectrometers. *Methods in molecular biology (Clifton, NJ).* 2017;1550:223-33.
86. Burger T. Gentle Introduction to the Statistical Foundations of False Discovery Rate in Quantitative Proteomics. *J Proteome Res.* 2018;17(1):12-22.
87. Mootha VK, et al. PGC-1alpha-responsive genes involved in oxidative phosphorylation are coordinately downregulated in human diabetes. *Nature genetics.* 2003;34(3):267-73.
88. Subramanian A, et al. Gene set enrichment analysis: a knowledge-based approach for interpreting genome-wide expression profiles. *Proceedings of the National Academy of Sciences of the United States of America.* 2005;102(43):15545-50.

89. Shannon P, et al. Cytoscape: a software environment for integrated models of biomolecular interaction networks. *Genome research.* 2003;13(11):2498-504.

90. Bindea G, et al. ClueGO: a Cytoscape plug-in to decipher functionally grouped gene ontology and pathway annotation networks. *Bioinformatics (Oxford, England).* 2009;25(8):1091-3.
