# Supplementary material for: KIBRA repairs synaptic plasticity and promotes resilience to tauopathy-related memory loss
Source: J Clin Invest. 2024 Feb 1;134(3):e169064. doi: 10.1172/JCI169064 (PMC10836803; doi:10.1172/JCI169064)

Full unedited gels for Figure 1

Blot #1a: anti-KIBRA

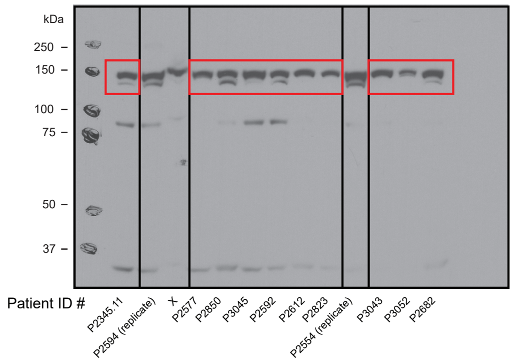

Blot #1b: anti-KIBRA

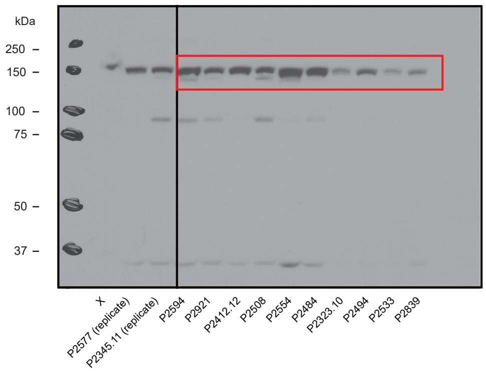

Blot #2a: MAb359, anti-tau acK274

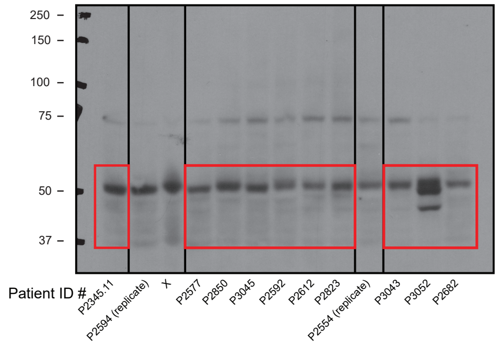

Blot #2b: MAb359, anti-tau acK274

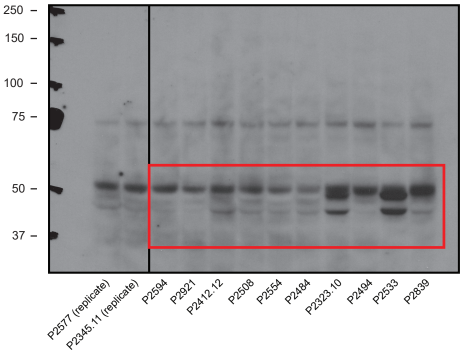

Blot #3a: AT270 anti-p-tau181

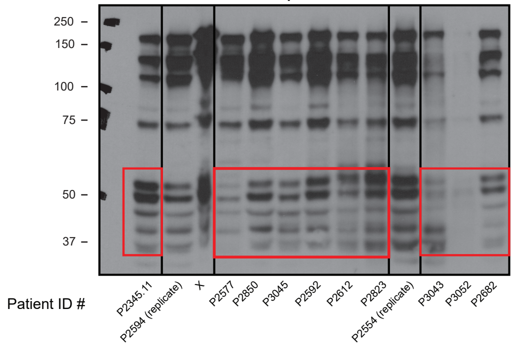

Blot #3b: AT270 anti-p-tau181

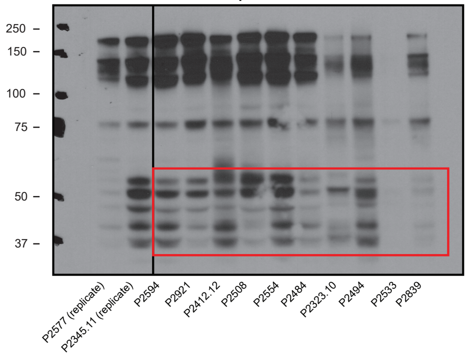

Blot #4a: HT7

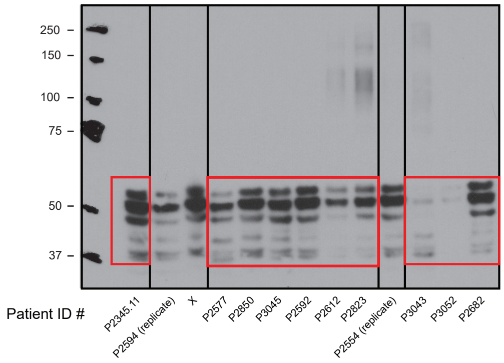

Blot #4b: HT7

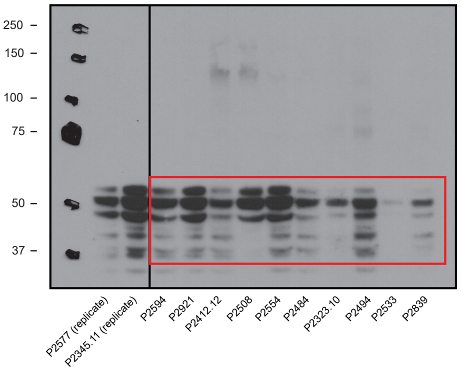

Blot #5a: anti-GAPDH

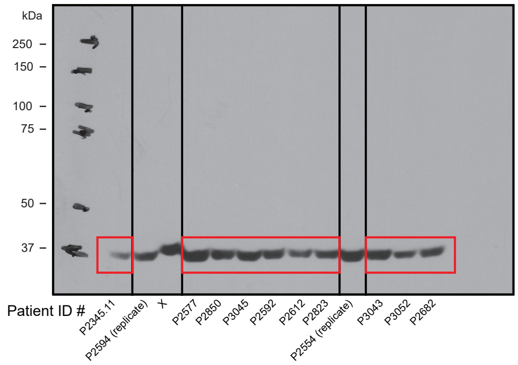

Blot #5b: anti-GAPDH

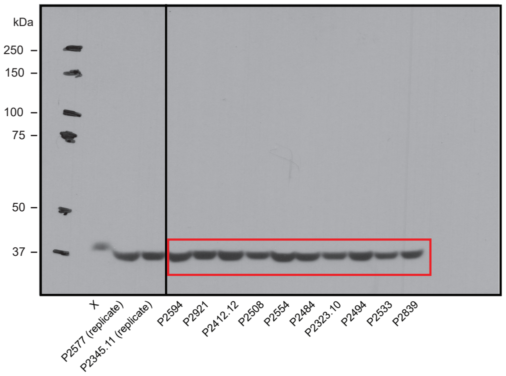

* Sample X: homogenate from one extra control brain, run in duplicate, was excluded from analyses because it displayed distorted, uneven, and inconsistent bands by western blotting.

Full unedited gels for Figure 4

Blot #1: AT180

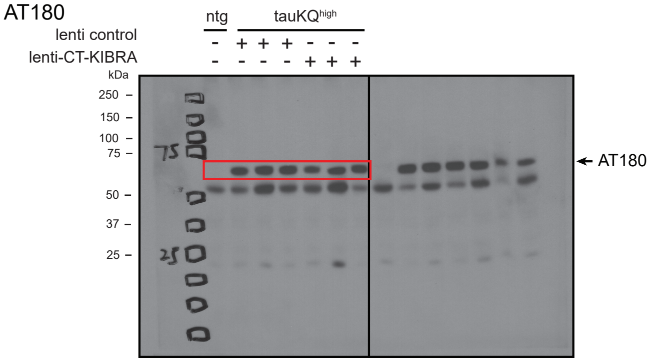

Blot #2: AT270

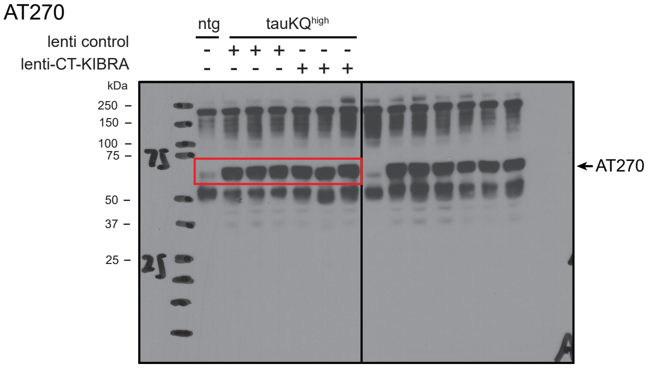

Blot #3: HT7

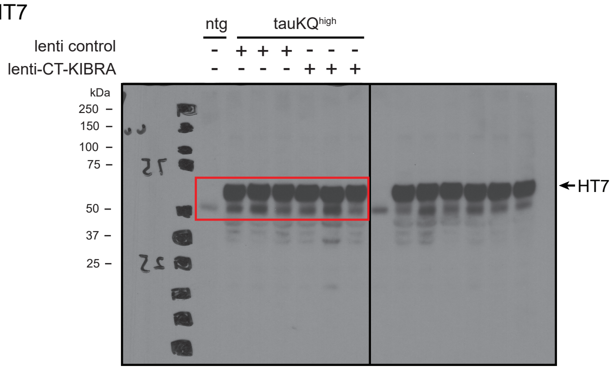

Blot #4: Tau5

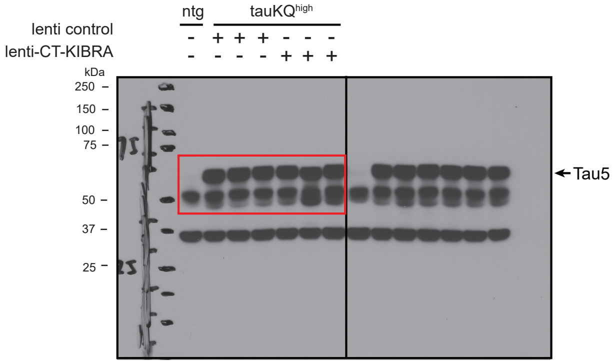

Blot #5: anti-GAPDH

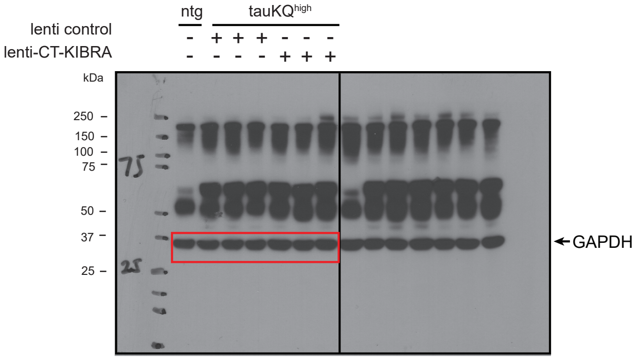

Full unedited gels for Figure 5

Blot #1: anti-PKMζ

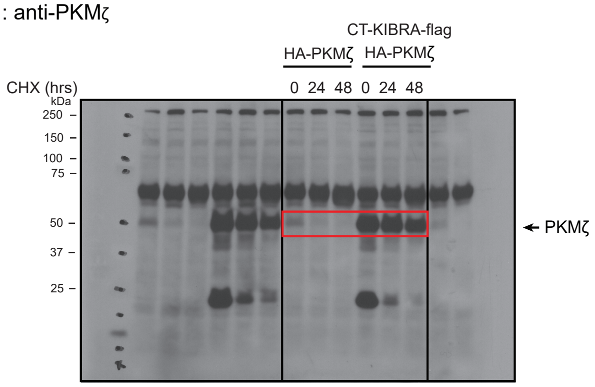

Blot #2: anti-flag

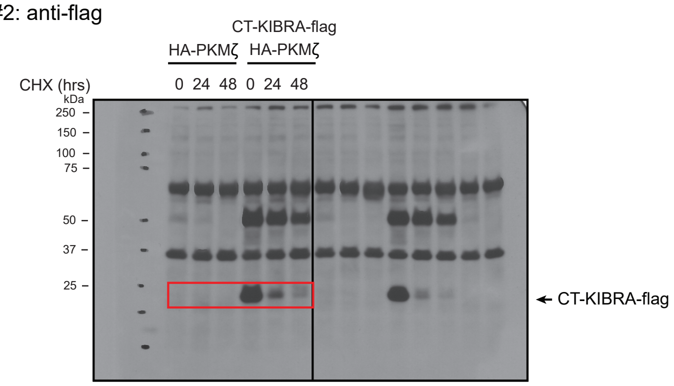

Blot #3: anti-GAPDH

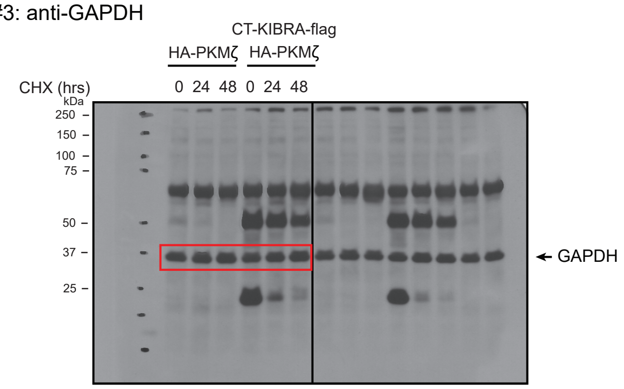

Full unedited gels for Figure 6

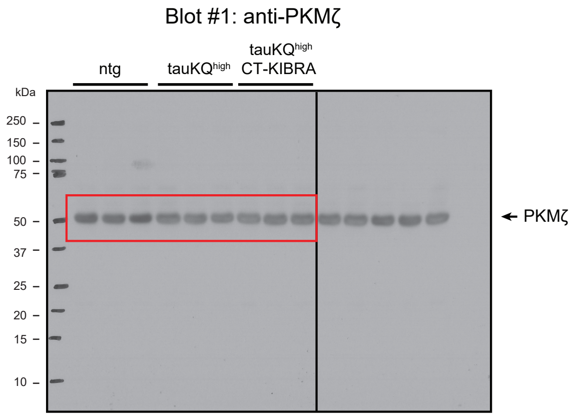

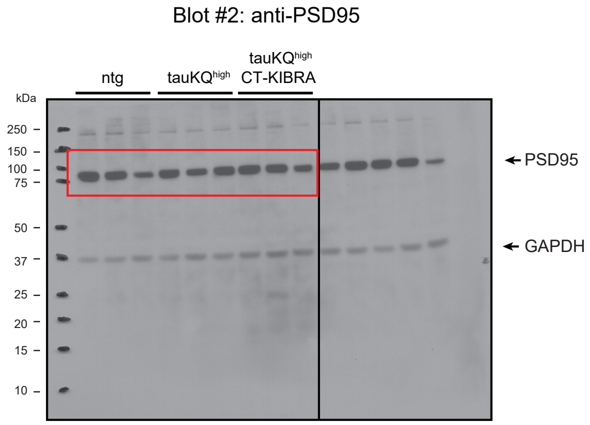

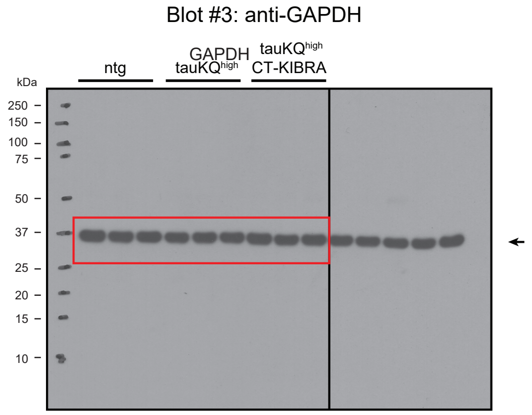

Full unedited gels for Supplemental Figure 3

Blot #1: anti-GluA2/3

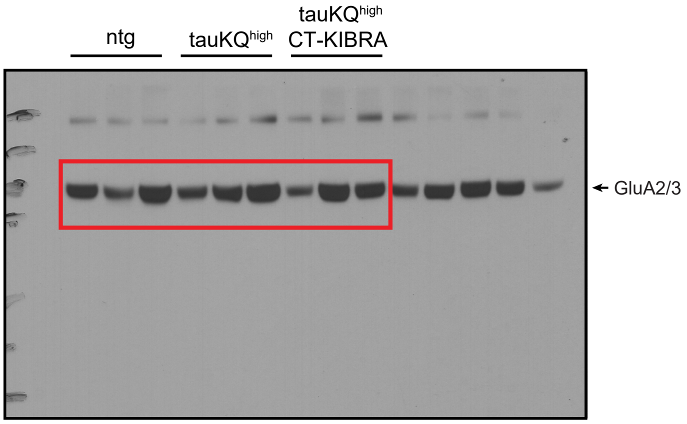

Blot #2: anti-Synaptotagmin-1

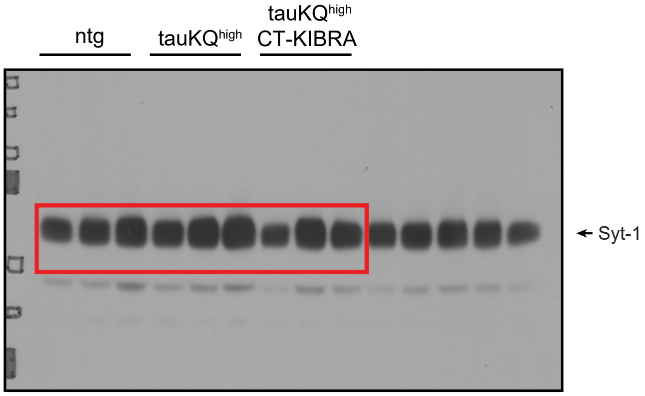

Blot #3: anti-GluN1

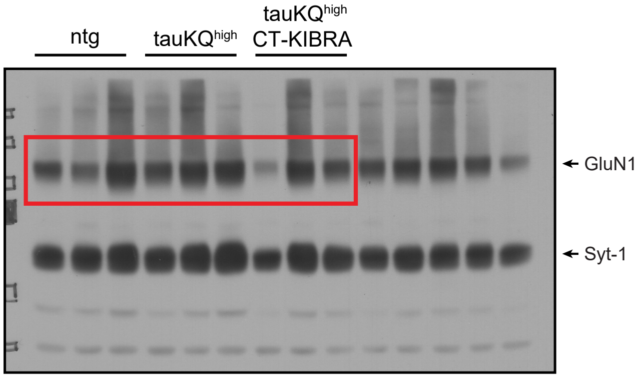

Blot #4: anti-GAPDH

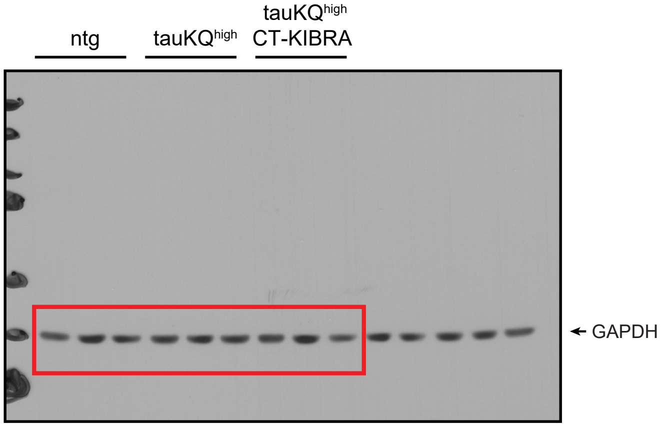

Full unedited gels for Supplemental Figure 5

Gel #1: PKMζ

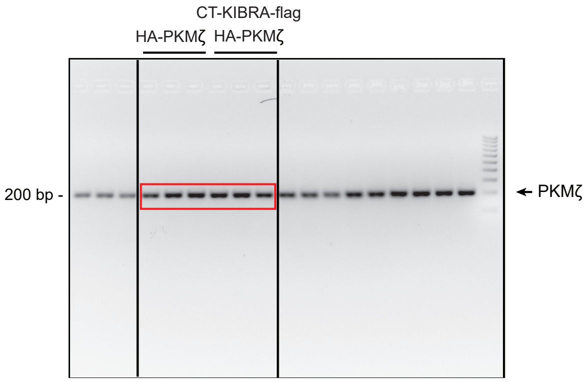

Gel #2: actin

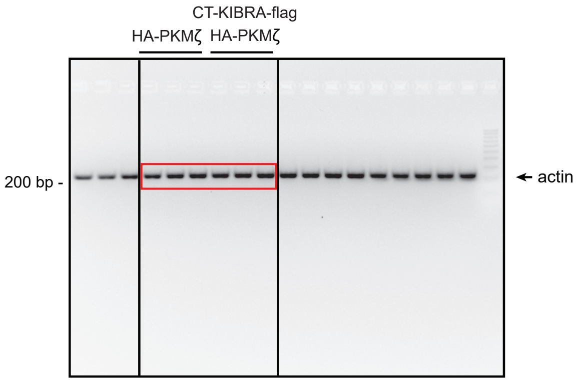

Full unedited gels for Supplemental Figure 6

A

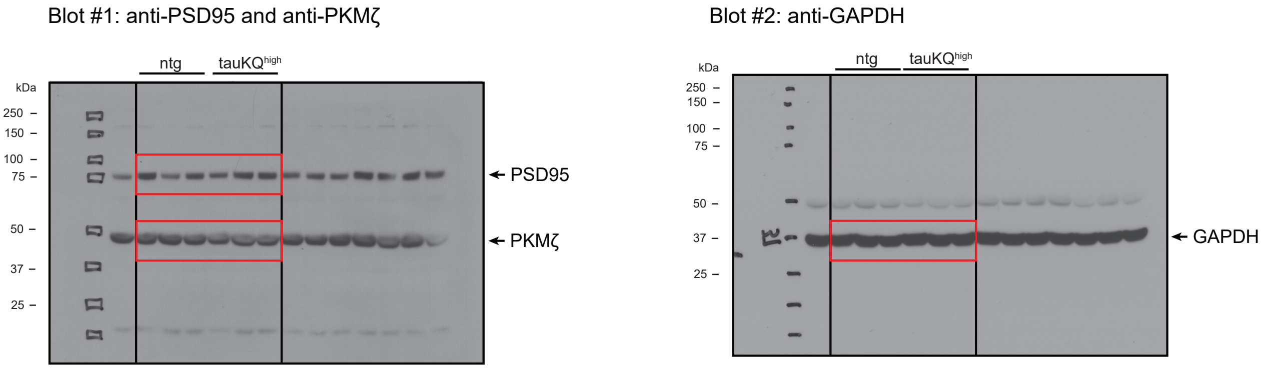

C

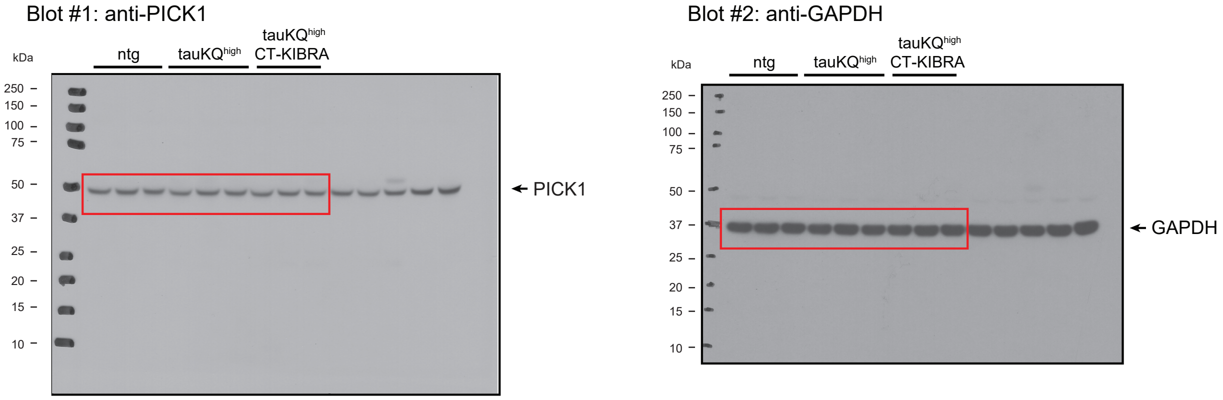

Supplement: Unedited blot and gel images [file jci-134-169064-s029.pdf]
